# Cell‐Free Fat Extract (CEFFE) as a Versatile Cell‐Free Biologic for Multitissue Regeneration: From Skin and Bone to Nerve, Reproductive, and Systemic Applications—A Comprehensive Review

**DOI:** 10.1155/bmri/9479492

**Published:** 2026-08-02

**Authors:** Arian Karimi Rouzbahani, Mahya Aliakbari, Bahar Amiri, Tahereh Fereydoonnezhad, Arya Behzadi, Somayeh Delfani, Zeinab Sharafi, Sepehr Miran, Abdolrazagh Marzban

**Affiliations:** ^1^ Razi Herbal Medicines Research Center, Lorestan University of Medical Sciences, Khorramabad, Iran, lums.ac.ir; ^2^ Surgery Education and Researching Network (SERGN), Universal Scientific Education and Research Network (USERN), Melbourne, Australia, usern.tums.ac.ir; ^3^ Student Research Committee, School of Medicine, Bushehr University of Medical Sciences, Bushehr, Iran, bpums.ac.ir; ^4^ Student Research Committee, Lorestan University of Medical Sciences, Khorramabad, Iran, lums.ac.ir; ^5^ Student Research Committee, Bushehr University of Medical Sciences, Bushehr, Iran, bpums.ac.ir; ^6^ School of Medicine, Shahid Beheshti University of Medical Sciences, Tehran, Iran, sbmu.ac.ir; ^7^ Department of Biochemistry, School of Medicine, Lorestan University of Medical Sciences, Khorramabad, Iran, lums.ac.ir; ^8^ Alfred Health, Department of Urology, Melbourne, Australia, alfred.org.au; ^9^ Department of Surgery, Monash University, Melbourne, Australia, monash.ac.za

**Keywords:** angiogenesis, cell-free fat extract (CEFFE), growth factors, reproductive health, tissue regeneration, wound healing

## Abstract

**Background:**

Cell‐free fat extract (CEFFE), an adipose‐derived biologic, offers a potent alternative to cell‐based therapies by delivering a complex milieu of growth factors (e.g., VEGF, EGF, and BDNF) and regulatory proteins without the tumorigenic, immunogenic, or ethical challenges associated with stem cell applications.

**Objective:**

The objective of this study is to systematically evaluate CEFFE′s regenerative efficacy across diverse tissue systems—including musculoskeletal, neural, reproductive, dermal, ocular, and systemic applications—while synthesizing its underlying molecular mechanisms, clinical outcomes, and translational challenges.

**Methods:**

A comprehensive literature review was conducted utilizing databases such as PubMed, Scopus, and Web of Science, focusing on preclinical and clinical studies published from January 2018 to May 2026. Over 55 articles were analyzed to evaluate CEFFE′s efficacy, dosage–response patterns, and molecular pathways.

**Results:**

CEFFE accelerates tissue repair through three conserved mechanistic axes: antiapoptotic survival (e.g., PI3K–Akt/mTOR activation and NRF2‐mediated antiferroptosis), context‐specific immunomodulation (e.g., Annexin A5–mediated M2 macrophage polarization and NLRP3 inflammasome disruption), and extracellular matrix remodeling alongside angiogenesis (e.g., TGF‐*β*/Smad signaling). Emerging evidence highlights CEFFE′s efficacy in novel domains such as intervertebral disc degeneration, corneal neurodegeneration, systemic sepsis, and as an IVF culture media supplement. Clinical trials confirm its safety and efficacy in dermatological applications (e.g., infraorbital aging and hyperpigmentation) and demonstrate superiority over standard treatments, such as hyaluronic acid, for early‐to‐mid‐stage osteoarthritis.

**Conclusions:**

CEFFE represents a highly versatile and precise cell‐free biologic capable of driving multitissue regeneration. To fully realize its clinical and commercial potential, future research must address donor variability by establishing standardized dosing protocols, implementing key‐molecule quantification for batch consistency, and conducting large‐scale, multicenter randomized controlled trials.

## 1. Introduction

Tissue regeneration continues to pose a considerable challenge in clinical medicine, especially in the repair of defects resulting from trauma, burns, congenital anomalies, or surgical resections. Tissue expansion, which was first used in 1957, is now a key method in reconstructive surgery. This technique entails the insertion of a silicone expander subcutaneously, which is progressively inflated to mechanically elongate surrounding tissue, thus producing supplementary skin for reconstruction [1]. Conventional tissue expansion is effective but has some drawbacks, such as long expansion times, high rates of complications like infection or expander extrusion, and poor tissue quality caused by ischemia and necrosis.

To address these limitations, novel adjunctive therapies have been formulated, utilizing the regenerative capabilities of adipose tissue. Cell‐free fat extract (CEFFE), obtained from mechanically processed adipose tissue, is a promising cell‐free alternative abundant in bioactive molecules, including growth factors such as vascular endothelial growth factor (VEGF), epidermal growth factor (EGF), and brain‐derived neurotrophic factor (BDNF). These components facilitate cell proliferation, angiogenesis, and extracellular matrix (ECM) synthesis and exhibit anti‐inflammatory properties, devoid of the immunogenicity, tumorigenicity, or ethical dilemmas associated with cell‐based therapies such as adipose‐derived stem cells (ADSCs) [2, 3]. The expansive field of regenerative medicine has progressively acknowledged the significant importance of immunomodulation in the process of tissue repair. For instance, biomaterials designed to both passively and actively engage macrophages—central regulators of the inflammatory and reparative processes—have demonstrated efficacy in restoring immune microenvironmental homeostasis and facilitating the regeneration of various injured tissues [4]. Innovative scaffold platforms, such as oxygen atom–concentrating short fibrous sponges, have been shown to regulate cellular respiration by enhancing cellular adaptation to oxygen. These advancements have resulted in superior outcomes in promoting angiogenesis and accelerating wound healing in complex environments, including chronic diabetic wounds [5]. Moreover, strategies utilizing electrospun fiber–based materials have been recognized as a significant category of immune engineering methodologies within the field of regenerative medicine. These approaches effectively modulate the behavior of macrophages and neutrophilrs via surface modification, drug loading, physicochemical design, and biological grafting, thereby promoting anti‐inflammatory phenotypes and enhancing tissue repair processes [6].

**Figure 1:**
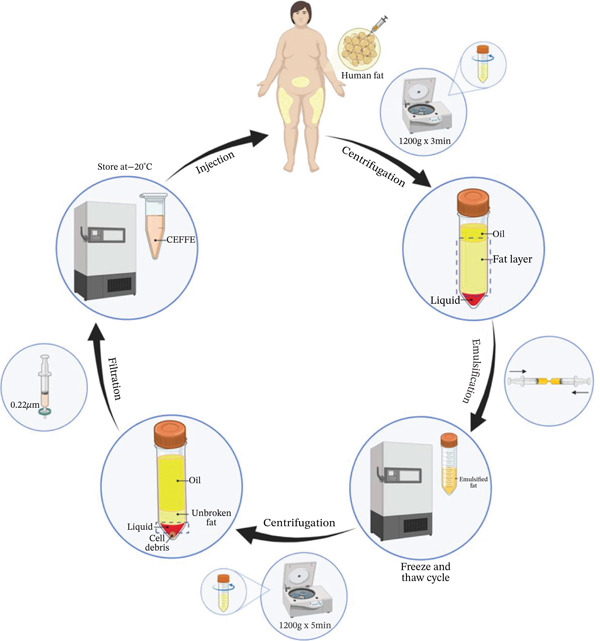
Preparation process of cell‐free fat extract (CEFFE) from human adipose tissue: This circular flowchart depicts the step‐by‐step extraction and processing of cell‐free fat extract (CEFFE) from human adipose tissue obtained via liposuction. Starting from the top, human fat is harvested and subjected to initial centrifugation at 1200 g for 3 min to separate into oil, a fat layer, and liquid debris. The unbroken fat is then emulsified using syringes connected by a three‐way valve. Subsequent centrifugation at 1200 g for 5 min removes unbroken fat, followed by a freeze–thaw cycle (freezing at −80°C and thawing at 37°C) to disrupt cells. Further centrifugation isolates the emulsified fat, and filtration through a 0.22‐*μ*m filter yields the final CEFFE, which is stored at −20°C or prepared for injection. The diagram emphasizes mechanical and thermal disruption to produce a growth factor–enriched, cell‐free liquid fraction suitable for regenerative applications.

A groundbreaking study conducted by Deng et al. in 2020 demonstrated CEFFE′s capacity to augment tissue expansion. In this study, 28 Wistar rats were allocated into control, low‐dose CEFFE (CEFFElow), and high‐dose CEFFE (CEFFEhigh) groups subsequent to expander implantation. CEFFE was made from human lipoaspirate by centrifugation, emulsification, freeze–thaw cycles, and filtration (as shown in Figure 1). It was then given under the skin. Over a period of 4 weeks, evaluations demonstrated diminished skin necrosis and retraction, augmented epidermal and dermal thickness, increased vascular density, and heightened cell proliferation markers in CEFFE‐treated groups relative to controls. These results demonstrate CEFFE′s capacity to enhance tissue viability and expansion efficiency via angiogenic and proliferative mechanisms [7].

CEFFE′s cell‐free composition serves as a complement to, and in certain instances a potential alternative for, cell‐based adipose methodologies. This includes cell‐supplemented autologous fat grafting strategies that have been investigated from laboratory settings to clinical applications aimed at enhancing fat graft retention and tissue regeneration through the incorporation of adipose‐derived stromal cells. Additionally, it presents an alternative to surgical nerve reconstruction techniques, such as regenerative peripheral nerve interfaces, which employ free autologous muscle grafts to biologically amplify peripheral nerve signals and mitigate neuroma formation in patients with amputations and nerve injuries [8, 9].

This review consolidates preclinical and clinical evidence regarding CEFFE′s applications in the regeneration of various tissues, including skin, bone, cartilage, nerves, and reproductive systems. We discuss the basic mechanisms, clinical applications, problems, and future directions to show how CEFFE can be used as a versatile and safe regenerative therapy.

## 2. Methods

This narrative review was undertaken to thoroughly assess the applications, mechanisms, and challenges of CEFFE in tissue regeneration. We used electronic databases like PubMed, Scopus, Web of Science, and Google Scholar to search for literature. The search strategy utilized keywords and MeSH terms including “cell‐free fat extract,” “CEFFE,” “tissue regeneration,” “wound healing,” “angiogenesis,” “growth factors,” “osteoarthritis,” “nerve regeneration,” “reproductive health,” “osteogenesis,” “bone tissue engineering,” “corneal repair,” “skin flap,” “sepsis,” “intervertebral disc degeneration,” “ferroptosis,” “IVF,” “diminished ovarian reserve,” “osteo‐angiogenic coupling,” and various combinations thereof (e.g., “CEFFE AND regeneration”). The search was restricted to articles published from January 2018 to May 2026 to emphasize recent developments, although seminal references from prior to this timeframe were incorporated for contextual relevance.

Inclusion criteria comprised original research articles, specifically preclinical studies (in vitro experiments and animal models), clinical trials, and systematic reviews utilizing CEFFE or comparable cell‐free adipose‐derived extracts for regenerative purposes. Research must provide information on efficacy, mechanisms, safety, or limitations. Non‐English language articles, duplicates, conference abstracts without full text, studies on unrelated fat‐derived therapies (e.g., whole adipose tissue or stem cell–only approaches), and those lacking empirical data (e.g., opinion pieces) were all reasons to leave something out.

We checked the titles and abstracts of the first searches, which yielded more than 350 records, for relevance. A full‐text review of 130 articles led to the selection of over 55 studies for in‐depth analysis. The substantially expanded literature base was achieved by broadening the search to include tissue applications not originally considered—including ocular surface repair, intervertebral disc degeneration (IVDD), bone–vascular coupling, flap surgery, IVF outcomes, prophylactic reproductive interventions, and systemic inflammatory conditions such as sepsis—extending the search to May 2026, and actively cross‐referencing bibliographies of included articles to identify relevant studies not captured in initial database searches. This expansion ensures comprehensive and representative coverage across CEFFE′s diverse regenerative applications spanning skin, bone, cartilage, nerve, disc, reproductive, ocular, vascular, and systemic tissues. Data extraction concentrated on study design, utilized models, principal outcomes (e.g., regeneration metrics and growth factor profiles), mechanisms (e.g., angiogenesis and anti‐inflammation), and constraints. Qualitative synthesis was utilized, incorporating thematic categorization based on tissue type and application. No formal meta‐analysis was conducted because of the variability in study designs and outcomes. The risk of bias was evaluated informally, considering study methodology, sample size, and controls, without employing a standardized tool, as this is a narrative review. The original articles reported that all of the studies that were included followed ethical rules, such as the ARRIVE guidelines for animal research and the CONSORT guidelines for clinical trials.

## 3. Applications of CEFFE in Tissue Regeneration

**Figure 2:**
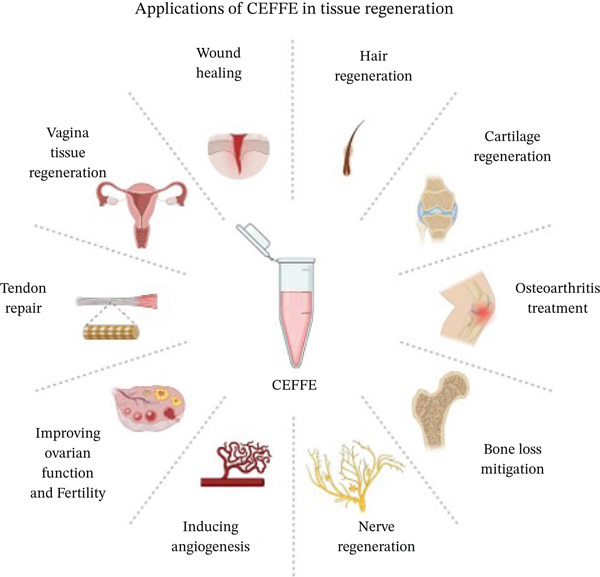
Applications of cell‐free fat extract (CEFFE) in tissue regeneration: This schematic illustrates the broad regenerative applications of cell‐free fat extract (CEFFE), depicted centrally as a vial containing the extract. Radiating lines connect to icons representing specific therapeutic areas: wound healing (depicted as a skin wound), hair regeneration (a hair follicle), cartilage regeneration (a joint structure), osteoarthritis treatment (an inflamed knee joint), bone loss mitigation (a bone cross‐section), nerve regeneration (branching neurons), inducing angiogenesis (a blood vessel network), improving ovarian function and fertility (an ovary with follicles), vaginal tissue regeneration (a uterus icon), and tendon repair (a tendon structure). The diagram emphasizes CEFFE′s versatility in promoting healing, angiogenesis, and functional restoration in preclinical and clinical contexts.

CEFFE is not only useful for tissue expansion, but it also has enormous potential for other uses in tissue regeneration, which could help different parts of the body. This section examines how CEFFE can aid tissue healing in different organs. Figure 2 summarizes the diverse applications of CEFFE across multiple tissue types, highlighting its potential in regenerative medicine.

## 4. Bone, Cartilage, and Tendons

A 2024 study published by Ding et al. explored the application of CEFFE‐loaded scaffolds for cartilage regeneration. The study utilized in vivo models, including nude mice for implantation, and in vitro models, such as human umbilical vein endothelial cells (HUVECs). CEFFE was extracted from the abdominal fat of six healthy female New Zealand white rabbits, approximately 2 months old and weighing 2.5 kg. Fresh adipose tissue was processed through centrifugation, emulsification, freezing at −80°C, thawing at 37°C, and filtration to obtain the third liquid layer. Using 3D printing, researchers prepared PLGA and CEFFE/PLGA scaffolds with nipple‐shaped trapezoidal columnar structures. They evaluated these scaffolds through histological staining (HE and Safranin O), biochemical analysis (GAG, hydroxyproline, and Collagen Type 2 [COL‐2]), and immunofluorescence (CD31 and COL‐2 expression). In vivo implantation into nude mice for 2 and 8 weeks revealed that the CEFFE/PLGA scaffolds promoted cartilage‐specific ECM deposition and formed more mature cartilage‐like tissue compared to the fibrous‐like structures in PLGA scaffolds. Mechanical testing and release kinetics analysis showed CEFFE release aligned with scaffold degradation. The outcomes demonstrated superior tissue retention, cell proliferation, and cartilage formation in CEFFE/PLGA scaffolds, making them promising candidates for cartilage repair. Future studies will address scaffold design optimization and long‐term performance evaluation [10].

Another study published in 2024 investigated the therapeutic potential of Annexin A5 (AnxA5), a key component of CEFFE, for osteoarthritis treatment. CEFFE was extracted from the abdominal or thigh adipose tissues of 10 healthy female volunteers aged 22–35. Researchers used in vitro models (mouse macrophages and chondrocytes) and an in vivo monosodium iodoacetate (MIA)–induced OA rat model. CEFFE underwent ion exchange chromatography for AnxA5 purification. In vitro, AnxA5 suppressed M1 macrophage polarization, reduced reactive oxygen species (ROS), and promoted TLR4 internalization and degradation. RNA sequencing revealed inhibition of the TLR signaling and NF‐*κ*B pathways. In vivo intra‐articular AnxA5 administration alleviated pain, reduced synovial inflammation, and protected cartilage.

CEFFE was applied at three concentrations (100, 250, and 500 *μ*g/mL) in vitro; cartilage structure scores on HE staining and Osteoarthritis Research Society International (OARSI) scores on Safranin O staining both declined in a concentration‐dependent manner, with the CEFFEhigh group showing the closest approach to normal control (*p* < 0.05; *n* = 6 per group). Histological analyses highlighted improved macrophage polarization and decreased cartilage damage in OA rats. This study underscores the need to explore other bioactive components in CEFFE, as its complexity (over 1700 proteins) presents fundamental challenges for identifying key therapeutic factors—a critical issue discussed in depth in the “Synergism Versus Key Molecule Contributions—Deconstructing CEFFE′s Therapeutic Complexity” section [11].

Another study conducted by Xu et al. in 2022 focused on the ability of CEFFE to mitigate bone loss caused by microgravity. CEFFE was extracted from the adipose tissue of healthy volunteers and tested on a tail suspension mouse model and the MLO‐Y4 osteocyte cell line. Researchers used techniques like *μ*CT, histology, immunohistochemistry, and apoptosis assays to evaluate outcomes. CEFFE treatment significantly restored bone volume, trabecular number, and cortical thickness in the tail suspension model. It also reduced ROS‐induced osteocyte apoptosis and cleaved Caspase‐3 expression while activating the ERK pathway. Histological assessments showed improved bone microarchitecture and reduced matrix degradation. Despite these promising results, the study acknowledged the need for longer term studies and additional research into CEFFE′s role in other osteoporosis causes [12].

In 2022, another study assessed the therapeutic effects of CEFFE on osteoarthritis using both in vitro and in vivo experiments. CEFFE was extracted from the adipose tissue of five healthy female volunteers and tested on MIA‐induced OA rat models and mouse macrophage and chondrocyte cell lines. CEFFE was found to alleviate pain and cartilage damage in OA rats. It promoted cartilage regeneration and angiogenesis, increased M2 macrophage polarization, and reduced proinflammatory markers (e.g., Interleukin‐1*β* [IL‐1*β*] and tumor necrosis factor‐*α* [TNF‐*α*]). Histological analysis revealed improved cartilage structure and increased capillary density in synovial tissue. While the study pointed out the possible benefits of CEFFE in OA treatment, it also called for further research into batch variability and unexplored CEFFE components, such as BDNF and GDNF [13].

Another study conducted by Kan in 2023 examined CEFFE‐loaded microneedles (CEFFE‐MNs) for treating Achilles tendinopathy. CEFFE was derived from the subcutaneous adipose tissue of healthy volunteers and encapsulated in a bioactive hydrogel to create MNs for transdermal delivery. Researchers evaluated the effects of CEFFE‐MNs on in vitro tendon cells and in vivo tendinopathy rat models. CEFFE‐MNs restored the mechanical strength of Achilles tendons, reduced apoptosis, and inhibited TNF signaling and mitochondrial dysfunction in tendon cells. Histological analyses showed decreased matrix degradation, improved mitochondrial morphology, and increased Col1 and Col3 expression. CEFFE‐MNs outperformed CEFFE injections in antiapoptotic and tendon repair effects. The study emphasized the importance of maintaining the biological activity of CEFFE in treatments, proposing microneedles as a promising delivery system. Challenges include ensuring long‐term efficacy and addressing variability in CEFFE properties [14].

A 2025 study by Li et al. extended the investigation of CEFFE‐functionalized electrospun fibers to focus specifically on osteoangiogenic coupling—the coordinated process by which bone formation and vascularization are simultaneously promoted, which is essential for effective regeneration of bone defects. Using polycaprolactone/gelatin (PCL/GT) fibers surface‐coated with polydopamine (PDA) to immobilize CEFFE (FE‐PDA@PCL/GT), the researchers employed a transwell coculture system to examine paracrine interactions between osteoblasts and endothelial cells. The release of CEFFE from FE‐PDA@PCL/GT fibers not only promoted osteogenesis and angiogenesis independently but also markedly enhanced reciprocal paracrine communications between endothelial cells and osteoblasts. This dynamic cell–cell interaction was identified as the key mechanism driving the observed enhancement of osteoangiogenic coupling. The engineered fiber constructs are envisioned as biomimicking scaffolds for vascularized bone defect repair, offering a dual‐function platform that simultaneously addresses bone matrix formation and adequate vasculature. Future validation in large‐animal bone defect models is needed to confirm clinical translatability [15].

A 2025 study by Nie et al. investigated whether CEFFE could enhance the osteogenic differentiation of ADSCs as a strategy for bone tissue engineering. Despite their accessibility, ADSCs have inherently limited osteogenic differentiation potential compared to bone marrow–derived counterparts. CEFFE was characterized biochemically, and ADSCs were treated with CEFFE and assessed for proliferation, calcium nodule formation, and osteogenic marker expression. An ADSC‐based osteogenic microtissue was created with CEFFE and implanted subcutaneously in nude mice to assess in vivo osteogenic activity. Transcriptomic analysis revealed that CEFFE upregulated genes associated with osteogenic differentiation and activated the PI3K–Akt signaling pathway, confirmed by Western blotting. CEFFE‐treated ADSCs demonstrated early formation of dense osteogenic cell sheets and significantly increased ectopic bone volume in vivo. These findings suggest that CEFFE may overcome a key limitation of ADSC‐based bone tissue engineering through a defined intracellular signaling mechanism [16].

### 4.1. Intervertebral Disc

A 2025 study published in *Biomedicines* investigated CEFFE as a novel therapeutic strategy for IVDD—one of the leading causes of low back pain globally. CEFFE was applied to nucleus pulposus cells (NPCs) in which degeneration was induced by TNF‐*α* and the oxidative stressor tBHP (tert‐butyl hydroperoxide) in vitro, and a rat caudal intervertebral disc puncture–induced degeneration model was used in vivo. CEFFE significantly reduced TNF‐*α*‐induced inflammation in NPCs by inhibiting the MAPK and NF‐*κ*B pathways and promoted matrix synthesis, as evidenced by restored COL‐2 levels. Critically, CEFFE activated the NRF2 transcription factor, thereby preventing tBHP‐induced ferroptosis—a newly recognized form of iron‐dependent oxidative cell death increasingly implicated in IVDD pathogenesis. CEFFE also scavenged excess ROS, reduced ferrous ion accumulation, and eliminated lipid peroxides in NPCs. In vivo, CEFFE‐treated rats showed notably higher disc preservation scores, reduced loss of nucleus pulposus material on Safranin O staining, and enhanced GPX4 expression, underscoring its efficacy in maintaining disc integrity and mitigating ferroptotic mechanisms. These findings represent the first demonstration of CEFFE′s application in spinal disc disease, establishing a new therapeutic frontier for CEFFE beyond musculoskeletal soft tissue and cartilage regeneration. Future studies should evaluate CEFFE delivery vehicles (e.g., injectable hydrogels) for sustained intradiscal release and investigate their long‐term effects in larger animal models [17].

## 5. Nerve, Angiogenesis, and Fat Grafts

After investigating the effects of CEFFE on bones and cartilage, this subsection investigates its role in nerve regeneration, angiogenesis, and the integration and survival of fat grafts.

A study conducted by Sun et al. in 2024 investigated the effects of CEFFE on axon regeneration and retinal ganglion cell (RGC) survival following optic nerve crush. CEFFE was derived from the middle‐fat layer of centrifuged adipose tissue obtained from healthy female donors undergoing liposuction. The tissue was mechanically emulsified, subjected to freeze–thaw cycles, and centrifuged to isolate the aqueous layer containing CEFFE. In this study, CEFFE was intravitreally injected into mice post–optic nerve crush, with BDNF serving as a positive control and PBS as a negative control. Researchers quantified axon regeneration at various distances from the crush site and assessed RGC survival. GO annotation revealed that 146 proteins in CEFFE were involved in axonogenesis and neurogenesis. CEFFE significantly promoted axon regeneration and RGC survival compared to controls. It acted on diverse pathways with high levels of inflammatory factors and modulated microglial activation in retinal tissue. Histological staining showed reduced neuroinflammation in CEFFE‐treated groups. The study highlighted the challenges of identifying the precise combination of factors within CEFFE responsible for its neuroprotective effects and recommended further research into its mechanisms of action [18].

Another study by Nie et al. in 2024 examined the role of CEFFE in enhancing the survival of high‐quality fat (HQF) grafts. CEFFE was derived from the fat remnants after isolating HQF from the lipoaspirates of healthy female donors. HQF grafts were transplanted into nude mice, with some groups receiving CEFFE treatment. The effects of CEFFE were also studied in vitro on ADSCs, HUVECs, and macrophages. CEFFE significantly improved HQF graft survival by promoting angiogenesis, reducing apoptosis, and enhancing cell proliferation. Transcriptomic analysis of ADSCs treated with CEFFE revealed 1684 differentially expressed genes, with many involved in angiogenesis. Histological analyses showed enhanced vascularization and proliferation in CEFFE‐treated grafts. The study proposed CEFFE as a promising tool for improving fat graft survival but noted challenges in elucidating its molecular pathways and the need for human clinical trials to confirm its efficacy [19].

A study led by Yu et al. in 2018 investigated the use of CEFFE in promoting angiogenesis in a mouse model of hindlimb ischemia. CEFFE was prepared from human adipose tissue using centrifugation and freeze–thaw cycles to isolate the liquid fraction. Mice with femoral artery ligation were treated with CEFFE, and blood perfusion was monitored using laser Doppler imaging. CEFFE significantly enhanced blood flow recovery, reduced tissue necrosis, and stimulated angiogenesis in ischemic tissues. Proteomics identified 1767 proteins in CEFFE, with 56 related to angiogenesis. In vitro assays demonstrated that CEFFE promoted proliferation, migration, and tube formation in HUVECs, while in vivo studies confirmed its angiogenic effects through increased capillary density. Challenges included donor variability and the transient nature of CEFFE′s effects, suggesting the need for multiple dosing strategies in clinical applications [20].

In 2024, Li et al. explored the use of CEFFE‐loaded fibers for promoting vascularization. Electrospun PCL/GT fibers were functionalized with PDA to enable CEFFE immobilization, creating CEFFE‐PDA@PCL/GT fibers. The fibers were tested in vitro on HUVECs, using the chick chorioallantoic membrane (CAM) assay, and in vivo in a rat subcutaneous embedding model. Results indicated that PDA‐coated fibers exhibited high CEFFE loading efficiency and sustained release. CEFFE‐PDA@PCL/GT fibers enhanced HUVEC proliferation, migration, and tube formation while promoting vascularization in vivo. Histological staining demonstrated improved microvascular development in implanted constructs. The study pointed out the advantages of CEFFE‐loaded fibers for therapeutic applications but noted the need for further optimization to enhance their vascularization capabilities [21].

Lastly, Zheng et al. in 2019 investigated the effects of CEFFE on fat graft survival using nude mice. CEFFE and nanofat were derived from human lipoaspirates and cotransplanted with macrofat grafts. CEFFE injections enhanced graft survival by promoting angiogenesis, reducing apoptosis, and improving proliferation in ADSCs and HUVECs. Synergistic effects of CEFFE and nanofat were observed, suggesting their combined use for superior fat graft outcomes. The study pointed out the future promise of CEFFE in regenerative medicine but stressed that further research is needed on its long‐term effects and clinical applications [22].

A 2025 study by Chen et al. investigated whether encapsulating CEFFE within a gelatin methacrylate (GelMA) hydrogel could overcome the major clinical limitation of rapid leakage and short duration of effectiveness associated with direct CEFFE injection. CEFFE derived from healthy volunteers was incorporated into GelMA to create a sustained‐release Ceffe@GelMA system. Extensive physicochemical characterization confirmed that Ceffe@GelMA retained a porous, injectable structure with sustained CEFFE release capability. In vitro, Ceffe@GelMA maintained CEFFE′s angiogenic and proliferative activities, significantly promoting HUVEC and L929 fibroblast migration, tube formation, and antioxidative stress resistance. In a murine random skin flap model, Ceffe@GelMA demonstrated marked prorepair activity, confirmed through thermal mapping, histological staining, and immunofluorescence for CD31, VEGF, and *α*‐SMA. This study represents a significant advance in CEFFE delivery strategy—overcoming instability and rapid clearance—and establishes a CEFFE‐incorporated hydrogel system as a clinically promising approach for flap necrosis prevention. Further molecular mechanistic investigation and large animal studies are still required [23].

### 5.1. Ocular Applications—CEFFE in Corneal Repair

A 2025 study by Xie et al. examined CEFFE′s therapeutic potential in corneal epithelial injury and corneal nerve degeneration—two unresolved challenges in ophthalmology. Two murine models were employed: a laser‐induced corneal neurodegeneration model and an alkali burn–induced corneal epithelial injury model. Topical CEFFE eye drops were administered and evaluated through comprehensive histological, molecular, and functional analyses alongside RNA sequencing. CEFFE significantly accelerated corneal wound closure and promoted epithelial cell proliferation, as confirmed by increased Ki67‐positive cells. Importantly, CEFFE preserved corneal nerve density and branching complexity and upregulated key neurotrophic factors critical for corneal nerve regeneration. Mechanistically, CEFFE exerted its dual epithelial and neural protective effects via IL‐1*β*/IL‐18‐mediated anti‐inflammatory and antiapoptotic pathways, with twice‐daily administration showing superior efficacy. This study positions CEFFE as a promising cell‐free topical therapy for ocular surface disorders, including neurotrophic keratopathy, and extends CEFFE′s application beyond injection‐based routes to topical ocular delivery—a clinically highly accessible and patient‐friendly route of administration [24].

## 6. Reproductive System

CEFFE may not only influence angiogenesis, neuronal growth, and tissue regeneration, but it could also affect the reproductive system.

In 2022, Liu et al. investigated the effects of CEFFE on ovarian function and fertility in aged mice. Specific pathogen‐free 10‐month‐old female mice were treated with 200 *μ*L of CEFFE (3 mg/mL) via tail vein every 2 days for 2 weeks, while control groups received saline injections. Human primary ovarian granulosa cells (hGCs) and KGN cells were also treated with CEFFE in vitro to evaluate cellular effects. The study employed methods such as histology, immunohistochemistry, TEM, RNA sequencing, and qRT‐PCR to assess ovarian health and gene expression. Results indicated that CEFFE treatment increased serum anti‐Müllerian hormone (AMH) and estradiol (E2) levels while reducing follicle‐stimulating hormone (FSH) levels, improved the number and quality of ovarian follicles, enhanced oocyte retrieval, and promoted embryonic development and litter size in aged mice. Additionally, CEFFE promoted granulosa cell proliferation, reduced senescence, and induced angiogenesis in the ovarian medulla. Although further studies are necessary to confirm its clinical relevance, the findings demonstrated CEFFE′s potential to restore ovarian function and improve fertility [25].

Another study conducted by Liu et al. in the same year investigated CEFFE′s effects on mice with chemotherapy‐induced premature ovarian insufficiency (POI). Female C57BL/6N mice were treated with 200 *μ*L of CEFFE (1.5 or 3 *μ*g/*μ*L) every 2 days for 2 weeks, while controls received PBS. Vaginal smears, follicle counts, and oocyte collection were performed alongside molecular analyses such as the TUNEL assay and RNA sequencing. Results demonstrated that CEFFE restored serum AMH, E2, and FSH levels, increased healthy follicle numbers, enhanced oocyte retrieval, and improved embryo development and litter size. In vitro, three concentrations of CEFFE (CF‐L: 0.06 *μ*g/*μ*L; CF‐M: 0.15 *μ*g/*μ*L; and CF‐H: 0.3 *μ*g/*μ*L) were tested on CTX‐injured KGN cells; CEFFE promoted KGN cell proliferation, improved mitochondrial function, and inhibited apoptosis in a concentration‐dependent pattern (*p* < 0.05). While these findings support CEFFE′s therapeutic potential for POI, further research is needed to address differences between induced and natural POI [25].

In 2022, Kang et al. evaluated CEFFE′s effects on vaginal tissue regeneration in perimenopausal and postmenopausal women and mice. CEFFE was administered topically using a 2% carbomer gel at concentrations of 25% and 50%. Histological analyses, hormone measurements, and Western blotting were used to assess outcomes. Results indicated that CEFFE improved vaginal epithelial thickness, increased epithelial layers, and enhanced protein expression of Ki‐67, ER‐*α*, PI3K, and AKT in vaginal epithelial cells. Hormone analyses revealed no significant differences in CEFFE composition between younger and older donors. The study highlighted CEFFE′s potential for addressing estrogen deficiency–related vaginal atrophy, though additional research is needed to confirm efficacy in humans [26].

In 2023, Xu et al. explored the use of CEFFE‐incorporated hydrogels (CF@Cu‐PEG) for endometrial regeneration in mice with intrauterine adhesions (IUAs). CEFFE was immobilized within a copper‐coordinated hydrogel and tested in vitro on human endometrial stromal cells (HESCs), human endometrial epithelial cells (HEECs), and HUVECs and in vivo in IUA mouse models. Methods included histology, Masson′s trichrome staining, and immunohistochemistry. CF@Cu‐PEG treatment enhanced cell proliferation, reduced fibrosis, and increased angiogenesis, leading to improved endometrial thickness and pregnancy outcomes. The hydrogel exhibited biocompatibility and antibacterial activity. This study demonstrated the potential of CEFFE‐loaded hydrogels for treating IUA, with further validation required in human trials [27].

A 2025 clinical study by Zhou et al.—the first of its kind—investigated whether CEFFE supplementation of IVF culture media could improve embryo development and clinical outcomes in older women (≥ 35 years) with a history of previous IVF failure due to poor embryo quality. This prospective open‐label clinical trial was conducted at the Reproductive Medical Centre of Ruijin Hospital, Shanghai, enrolling patients between January 2022 and March 2023 with follow‐up through December 2023. CEFFE was derived from healthy donors and characterized for growth factor content. In vitro, CEFFE supplementation significantly improved mouse embryo development kinetics—accelerating cleavage times, increasing blastocyst rates, and reducing blastocyst apoptosis. In the clinical trial, CEFFE‐supplemented media enhanced embryo adhesion and invasion in endometrial coculture models. Importantly, CEFFE treatment was associated with higher pregnancy rates, reduced abnormal pregnancies, and increased live birth rates in this challenging patient population. This study represents a landmark advance, demonstrating that CEFFE can function not only as a tissue regenerative agent but also as a culture media supplement in assisted reproductive technology, opening an entirely new clinical application domain for CEFFE [28].

## 7. Skin and Hair Regeneration

**Figure 3:**
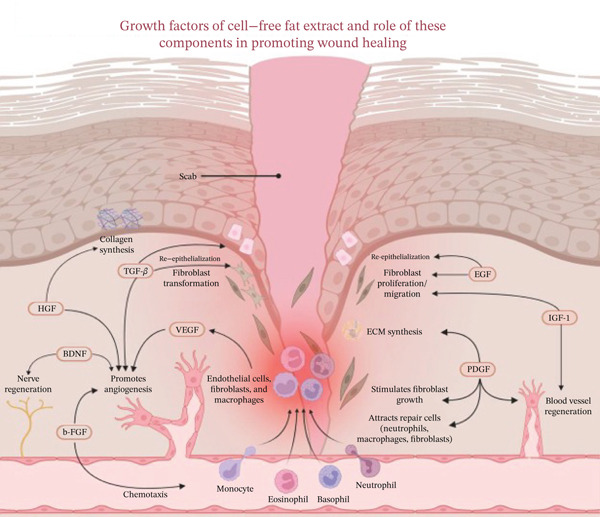
Growth factors of cell‐free fat extract and their role in promoting wound healing: This schematic illustrates the key growth factors present in cell‐free fat extract (CEFFE) and their contributions to the wound healing process. A cross‐section of a wound with a scab is depicted, showing cellular components such as endothelial cells, fibroblasts, macrophages, monocytes, eosinophils, neutrophils, and nerve endings. Growth factors including transforming growth factor‐*β* (TGF‐*β*) (promoting collagen synthesis and re‐epithelialization), FGF (e.g., basic fibroblast growth factor [bFGF] for chemotaxis and nerve regeneration), VEGF (promoting angiogenesis), BDNF (promoting angiogenesis), EGF (promoting fibroblast proliferation/migration and re‐epithelialization), Insulin‐Like Growth Factor 1 (IGF‐1) (promoting ECM synthesis), hepatocyte growth factor (HGF) (promoting nerve regeneration and angiogenesis), and platelet‐derived growth factor (PDGF) (stimulating fibroblast growth, attracting repair cells like neutrophils/macrophages/fibroblasts, and promoting blood vessel regeneration) are labeled with arrows indicating their specific roles in processes like fibroblast transformation, endothelial cell stimulation, ECM synthesis, and overall tissue repair.

CEFFE has demonstrated significant potential in enhancing skin health, wound healing, and hair regeneration. For wound healing, CEFFE accelerates tissue repair by promoting re‐epithelialization, collagen secretion, and angiogenesis while reducing inflammatory macrophage infiltration. It supports the proliferation and migration of keratinocytes and enhances endothelial cell functions, making it particularly promising for diabetic wounds and tissue regeneration. In skin rejuvenation, CEFFE enhances dermal thickness by stimulating angiogenesis and ECM production, offering protection against UVB‐induced photoaging and reducing ROS. Additionally, CEFFE′s potential in hair regeneration lies in its ability to regulate dihydrotestosterone (DHT)/ androgen receptor (AR) signaling and improve angiogenic conditions, addressing androgenetic alopecia (AGA) effectively. Beyond these applications, CEFFE has shown anti‐inflammatory properties, alleviating conditions like atopic dermatitis (AD) and rosacea by modulating immune responses and reducing proinflammatory markers. It is also being explored for applications such as fat graft survival and mitigating osteoarthritis. Figure 3 illustrates how these growth factors in CEFFE contribute to wound healing.

## 8. Preclinical and Clinical Studies on Skin and Hair Regeneration

This subsection details the mechanisms, methods, and results from studies showing how CEFFE could be used to treat people.

In 2022, Zhang et al. systematically evaluated the efficacy of CEFFE and platelet‐rich plasma (PRP) in facilitating wound healing. The study employed both in vitro models, such as human fibroblasts and HUVECs, and in vivo models, specifically 8‐week‐old BALB/c mice. CEFFE and PRP were made from healthy people′s fat and blood, respectively. CEFFE was made by centrifugation, mechanical emulsification, freezing and thawing, and filtration. PRP was made by centrifuging blood and collecting plasma fractions. BCA assays and ELISA were used to measure the levels of proteins and growth factors like BDNF, bFGF, HGF, EGF, NT‐3, TGF‐*β*, PDGF‐BB, and VEGF. CCK‐8, scratch wound, and tube formation tests were used to look at cell growth, movement, and angiogenesis. In vivo, full‐thickness wounds in mice were treated with subcutaneous injections of CEFFE, PRP, or PBS, and healing progress was monitored over 12 days. The results indicated that PRP had about 19 times more total protein than CEFFE, but the growth factor profiles were similar, with CEFFE having slightly higher NT‐3 levels. Both CEFFE and PRP greatly increased the growth of fibroblasts, the movement of cells, and the formation of tubes. There were no significant differences between the two. In vivo, both treatments significantly expedited wound closure and enhanced histological indicators of healing, including re‐epithelialization, collagen deposition, cell proliferation (Ki67), and angiogenesis (CD31), in comparison to controls. One issue was that CEFFE′s light red color was unclear, possibly due to carotenoids. The study raises the importance of CEFFE as a feasible alternative to PRP in wound healing, necessitating further investigation of its mechanisms [29].

In 2023, Cai et al. conducted a study on 15 female patients aged 18–33 years who had postradiotherapy pigmentation that did not respond to standard treatments such as topical medications and laser therapies. CEFFE was extracted from fat tissue obtained through liposuction. The preparation process included washing, centrifugation, emulsification, separation, filtration, and storage at −80°C. Each patient received five intradermal injections of CEFFE every 2 weeks, and their progress was checked at the start of treatment, 3 months later, 6 months later, and 12 months later. The study indicated that CEFFE treatment made a big difference in PIH. Objective outcomes comprised decreases in the brown spot (BS) index, increased skin lightness, enhanced lesion color, and diminished transepidermal water loss (TEWL), signifying improved skin barrier function. A total of 93.33% of patients reported “*improved*” or “*very much improved*” results on the Global Aesthetic Improvement Scale (GAIS). According to the Likert Satisfaction Scale (LSS), 100% of patients were “*satisfied*” or “*very satisfied*” with the treatment. When it developed tolerance, 53.33% of patients had temporary bruises, and all of them felt stinging during the injection. Nonetheless, no significant complications or withdrawals were reported. The study acknowledged challenges despite its promising findings, such as the limited sample size and the difficulty in pinpointing specific active components within CEFFE. Additional research involving larger cohorts and the investigation of CEFFE′s efficacy for other hyperpigmentation disorders is advised to corroborate and expand these findings [30].

Kang et al. conducted a study in 2024 to assess the effectiveness and safety of CEFFE in the treatment of infraorbital dark circles. The study integrated experimental and clinical methodologies, employing zebrafish embryos to examine CEFFE′s antimelanogenic properties and executing a single‐arm clinical trial involving 11 human subjects with infraorbital dark circles. CEFFE was made from fat taken from the lower abdomen of patients through liposuction. The extraction process included washing, centrifugation, emulsification, and filtration. This made a light pink liquid that was then put into containers and kept at −80°C. The clinical trial consisted of five intradermal injections of autologous CEFFE given every 2 weeks, with follow‐ups taking place 3 months after the last treatment. The zebrafish embryo model demonstrated a substantial decrease in melanin levels in CEFFE‐treated embryos relative to controls, suggesting an inhibitory effect on melanin synthesis. In the clinical trial, both subjective and objective evaluations confirmed the efficacy of CEFFE. More than 72% of patients said the treatment worked, and more than half said they were happy with it. At the 3‐month follow‐up, objective measurements showed significant changes in the color of the skin under the eyes and the wrinkles around the eyes, especially on the dark circles′ outer edges. There were only a few minor side effects, such as bruising, erythema, and edema, that went away on their own. The study′s findings were encouraging; however, limitations included the small sample size and the necessity for additional investigation into CEFFE′s active components and mechanisms. The researchers suggested the necessity for more extensive studies with prolonged follow‐up durations and enhanced objective evaluations to corroborate these findings and improve treatment protocols [31].

Wang et al.′s 2020 study looked at how CEFFE affected wound healing in a mouse model of diabetes. The study used male C57BL/KsJ db/db mice, a conventional model for Type 2 diabetes, to assess how effective CEFFE is in mitigating the delayed wound healing often associated with diabetes. CEFFE was made from human fat tissue taken from healthy women who had liposuction. The process of making the product involved washing, centrifugation, mechanical emulsification, freeze–thaw cycles, and filtration to obtain the aqueous CEFFE layer, which was then kept at −80°C. The mice were split into three groups: one that got a lot of CEFFE (CEFFEhigh), one that got a little CEFFE (CEFFElow), and one that did not acquire any CEFFE at all (PBS). A circular full‐thickness wound was inflicted on the central dorsal area of each mouse, followed by subcutaneous administration of CEFFE. The study indicated that CEFFE sped up wound healing in diabetic mice by a lot, as shown by the fact that the healing times were shorter. Specifically, healing time was reduced from 22.00 ± 2.00 days in the PBS control group to 18.00 ± 1.58 days in the CEFFElow group and 14.80 ± 1.09 days in the CEFFEhigh group (*p* < 0.01; *n* = 6 per group), indicating a clear dose–response relationship in which a higher CEFFE dose was associated with significantly faster wound closure. Histological analyses (HE staining, Masson′s trichrome staining, and CD31+ staining for angiogenesis) showed that CEFFE‐treated wounds had better re‐epithelialization, more collagen deposition, and more capillaries. CEFFE‐treated groups showed a big decline in the number of inflammatory macrophages (CD68+ cells), which shows that the treatment had an anti‐inflammatory effect. In vitro experiments corroborated these findings, demonstrating that CEFFE facilitates the proliferation and migration of human immortal keratinocyte cells (HaCaT) and enhances tube formation in human vascular endothelial cells (HUVEC). Western blot analysis corroborated augmented collagen fiber synthesis in CEFFE‐treated wounds. The research underscores CEFFE′s potential as a therapeutic agent for diabetic wound healing while also pinpointing avenues for future investigation. These encompass the clarification of the principal factors and signaling pathways implicated in CEFFE′s wound‐healing properties, as well as the examination of nerve regeneration in wounds treated with it. This study lays a promising foundation for additional investigation of CEFFE in wound healing, especially for individuals with diabetes [32].

Deng et al.′s study looked into how CEFFE affects tissue regeneration in a model that grows rat tissue. The research employed 4‐week‐old Wistar female rats for in vivo experiments and a human keratinocyte cell line (HaCaT) for in vitro analyses. CEFFE was made from human fat tissue that was taken from a person who had liposuction and gave their permission. The extraction process included washing, centrifugation, mechanical emulsification, and storage at −80°C. A bicinchoninic acid assay was used to find out how much protein was in the sample. There were three groups of rats: a control group (no treatment), a CEFFElow group, and a CEFFEhigh group. After the implantation of a tissue expander, the CEFFElow and CEFFEhigh groups received subcutaneous injections of 300 or 600 *μ*g of CEFFE protein, respectively. Every other day for 4 weeks, physiological saline was injected into the expander. The study indicated that CEFFE made tissue regeneration in the expanded skin much better. The main results were a lower rate of necrosis and a retraction of expanded skin, a thicker epidermis and dermis, a higher density of blood vessels, and a faster rate of cell growth. Protein expression analyses demonstrated elevated levels of VEGFR, EGFR, Collagen Type 1 (COL‐1), and Collagen Type 3 (COL‐3) in CEFFE‐treated skin, signifying improved vascularization and collagen synthesis. In vitro, CEFFE facilitated HaCaT cell proliferation and expedited the cell cycle by augmenting the fraction of cells in the S phase and G2/M phase. The study found no significant difference in how well CEFFE worked between low and high doses, which means that a lower dose is enough for effective tissue expansion. Histological analyses verified augmented epidermal and dermal thickness, heightened dermal collagen deposition, and an increased quantity of blood vessels and proliferating cells (PCNA‐positive) in CEFFE‐treated cohorts. The study recognized difficulties, such as the necessity to delineate the specific functional components of CEFFE and ascertain potential variability in therapeutic effects contingent upon donor sources. These results show that CEFFE could help tissues heal, but more research is needed to ensure it works well in the clinic [7].

The 2023 study examined the efficacy of CEFFE as a therapeutic intervention for AGA. The study utilized a DHT‐induced AGA mouse model and human dermal papilla cells (hDPCs) in vitro to assess the effects of CEFFE on hair regrowth, angiogenesis, and cellular activity. CEFFE was extracted from adipose tissue acquired through liposuction from healthy adult females and subsequently processed through washing, centrifugation, emulsification, filtration, and freezing. The mice were split into five groups: control, model, CEFFElow, CEFFEmiddle, and CEFFEhigh. Over the course of 16 days, their hair growth was measured using photos, histological tests, and immunohistochemistry. The findings demonstrated that CEFFE markedly elevated the anagen entry rate and hair coverage percentage, facilitated angiogenesis characterized by increased CD31‐positive capillary density, and augmented follicular cell proliferation as evidenced by Ki67 staining. In vitro, CEFFE counteracted DHT‐induced cell cycle arrest, inhibited apoptosis, diminished intracellular DHT concentrations, and downregulated AR expression, thereby facilitating hDPC proliferation. In vitro, CEFFE at 250 *μ*g/mL demonstrated the best therapeutic rescue of hDPCs from DHT (100 *μ*M)‐induced stress—outperforming both lower (50 *μ*g/mL) and higher (500 *μ*g/mL) concentrations—suggesting a nonlinear, optimum‐concentration relationship rather than a simple dose–response pattern. The study showed that CEFFE could be a beneficial noninvasive treatment for AGA, even though higher concentrations did not always work better. Some of the problems are figuring out which CEFFE parts are which, finding the best clinical doses, and figuring out how donor characteristics affect its composition. Future research ought to concentrate on comparative studies with current treatments and the clinical validation of CEFFE′s efficacy [33].

The research conducted in 2024 investigated the therapeutic efficacy of CEFFE in the treatment of AD. The study utilized in vivo models, such as female BALB/c mice and a DNCB‐induced AD mouse model, alongside in vitro investigations on HaCat cells and HUVECs, to examine the effects of CEFFE on oxidative stress, inflammation, and the restoration of the skin barrier. The protein concentration of CEFFE was measured using a BCA assay. It was made from washed and emulsified adipose tissue that had been frozen and thawed, centrifuged, and filtered. CEFFE exhibited antioxidant and anti‐inflammatory effects, diminishing ROS levels both in vitro and in vivo, while enhancing cell viability and apoptosis in HaCat cells subjected to oxidative stress. In the DNCB‐induced AD model, CEFFE mitigated AD symptoms by diminishing ′TEWL, reinstating epidermal thickness, and decreasing mast cell infiltration. CEFFE also lowered levels of inflammatory cytokines, like IL‐4, IL‐13, and IFN‐*γ*, and stopped CD4+ naïve T cells from turning into Th2 cells, bringing the Th1/Th2 balance back to normal. Immunofluorescence staining showed that CEFFE‐treated groups had less oxidative DNA damage (8‐OHdG). In the H_2_O_2_‐induced skin inflammation model, CEFFE decreased lesion areas, with elevated doses yielding superior results, including diminished epidermal thickness and expedited healing. Although CEFFE exhibited encouraging outcomes, the study highlighted apprehensions regarding the cytotoxicity associated with elevated CEFFE concentrations and discussed the necessity for standardized, safe, and effective dosing for commercial utilization. This research endorses CEFFE as a prospective therapeutic agent for AD, necessitating further studies to enhance its safety and efficacy for clinical application [34].

The 2024 study assessed the effectiveness and safety of CEFFE in treating infraorbital aging. This clinical study comprised 10 healthy female participants aged 31–58 years, all demonstrating signs of infraorbital aging with a Wrinkle Severity Rating Scale (WSRS) of 2 or higher. CEFFE was made from fat tissue taken from the abdomen through liposuction. The tissue was then washed, centrifuged, emulsified, and filtered to obtain a pure extract, which was kept at −20°C. Five infraorbital CEFFE injections were given to each participant every 2 weeks, and follow‐up assessments were done at 3, 6, and 12 months after the treatment. 2D photography, skin texture parameters (SEr, SEsm, and SEw), skin elasticity indices (R2, R5, and R7), and TEWL were used to measure objective outcomes. The GAIS and the LSS were used for subjective evaluations. Results indicated that CEFFE made a big difference in skin texture by making it smoother and less wrinkled. The changes were especially noticeable after 3 months. Skin elasticity improved a lot, reaching its peak at 6 months, and TEWL went down steadily, showing that the skin barrier was working better and holding onto moisture better. Seventy percent of participants said they were satisfied, and the average GAIS scores were 3.60 ± 0.35 (physician‐assessed) and 3.70 ± 0.48 (patient‐assessed). At the 12‐month follow‐up, WSRS scores went from 3.50 ± 0.67 to 2.60 ± 0.67. The study identifies CEFFE as a potentially effective treatment for infraorbital aging; however, it is constrained by a limited sample size, an exclusive focus on female participants, and a follow‐up duration of only 12 months. Future studies ought to investigate enduring effects, refine injection methodologies, and evaluate the prospective advantages of integrating CEFFE with alternative treatments, including hyaluronic acid (HA). These results indicate that CEFFE provides a secure and efficacious method for enhancing under‐eye aesthetics; however, larger and more varied studies are required for wider implementation [35].

A 2019 study investigated the efficacy of CEFFE in alleviating UVB‐induced photoaging via its antioxidant, antiapoptotic, and proangiogenic properties. The study evaluated the effects of CEFFE on skin rejuvenation utilizing both in vivo models comprising 6‐week‐old female BALB/c nude mice and in vitro models involving human dermal fibroblasts. CEFFE was isolated from adipose tissue acquired through lipoaspiration from healthy female donors. The extraction process involved washing, centrifugation, mechanical emulsification, and storage at −80°C. In vitro, fibroblasts were pretreated with CEFFE for 24 h prior to UVB exposure, and various outcomes, including ROS levels, cell proliferation, senescence, and protein expression (GPX1, SOD‐1, SOD‐2, catalase, and COL‐1), were assessed. In vivo, UVB‐irradiated mouse dorsal skin underwent treatment with CEFFE for 8 weeks, during which dermal thickness, capillary density, apoptotic cell count, and protein expression were evaluated. The findings indicated that CEFFE markedly decreased ROS levels, enhanced cell proliferation, and alleviated UVB‐induced cell cycle arrest and senescence in fibroblasts. CEFFE also increased the levels of antioxidant enzymes (GPX1, SOD‐1, and SOD‐2) and COL‐1 in both in vitro and in vivo. In the murine model, CEFFE treatment enhanced dermal thickness, elevated capillary density, and diminished apoptotic cell counts in UVB‐exposed skin. These results show how CEFFE can protect against photoaging and how it might help skin health and make it look younger. Even though the results were promising, the study brought up several problems. Further research is necessary to fully understand the mechanisms by which CEFFE regulates antioxidant enzyme expression, particularly through pathways like Nrf2 and Sirt1/FOXO. The study also recommended investigating the quality control of CEFFE preparation and the prospective advantages of utilizing CEFFE from various donor populations, including males. In general, this study shows that CEFFE could be a beneficial choice for skin rejuvenation therapies, but more research is needed to figure out how to use it best and how it works at the molecular level [36].

The 2024 study examined the therapeutic efficacy of recombinant human AnxA5 in the context of diabetic wound healing. The study assessed A5′s capacity to modulate inflammation, epithelialization, and tissue repair utilizing in vivo diabetic mouse models (BKS‐Leprem2Cd479/Gpt) and in vitro experiments with diverse cell types. A5 was added to GelMA hydrogels, put on wounds, and cross‐linked with UV light for localized treatment. The study demonstrated that A5 markedly expedited wound healing in diabetic mice relative to control and GelMA‐only groups, attaining near‐complete healing by Day 14. A5 modulated inflammation by facilitating macrophage polarization from the proinflammatory M1 phenotype to the prorepair M2 phenotype, as demonstrated by diminished M1 markers (CD86, IL‐1*β*, IL‐6, and TNF‐*α*) and elevated M2 markers (CD206 and TGF‐*β*). A5 increased the movement of epithelial cells in vitro, but it did not change how fast they grew. It also did not have much of an effect on angiogenesis or the regeneration of the ECM by dermal fibroblasts. A5 also stopped endothelial cells from forming tubes while encouraging them to move. The histological analysis corroborated these findings, demonstrating a continuous epidermis, preserved skin architecture, organized collagen configuration, and diminished inflammatory cell infiltration in A5‐treated wounds. The study observed that A5 treatment alone did not result in complete healing by Day 14, in contrast to CEFFE, indicating the necessity for additional therapeutic factors to facilitate angiogenesis and cell proliferation. The study indicated that additional research is necessary to refine A5′s concentration, dosing regimen, and mechanisms of action, especially its function in promoting epithelial cell migration. This study emphasizes A5′s potential as a targeted therapeutic agent for nonhealing wounds, especially in mitigating inflammation and promoting epithelialization [11].

The 2024 study examined the effectiveness of CEFFE and platelet‐rich fibrin (PRF) in enhancing scar maturation. The study utilized a rabbit ear hypertrophic scar model involving 12 New Zealand white rabbits to evaluate the effects of CEFFE, PRF, and their combination on scar formation and remodeling. CEFFE was derived from adipose tissue extracted from the rabbits′ inguinal regions, whereas PRF was produced by centrifuging rabbit blood to separate fibrinogen‐rich clots. The rabbits were split into four groups: CEFFE+PRF, CEFFE only, PRF only, and a control group that got saline. Histological analysis and the scar elevation index (SEI) were used to look at the results on Day 40 after the treatments were injected into the scar sites. The results indicated that the CEFFE+PRF combination was much better at stopping the formation of pathological scars than the other groups. This group showed the least amount of collagen buildup, the thinnest epidermis and dermis, a more organized collagen structure, and fewer inflammatory cells. Histological staining showed that the CEFFE+PRF group had regular collagen remodeling and fewer fibroblasts and capillaries. The SEI score for this group was the lowest (1.75 ± 0.03), which means that the scar was less noticeable. CEFFE and PRF alone also showed some improvements, but the combination therapy was better because it helped with adipogenesis and remodeling the ECM. The study underscored the therapeutic efficacy of CEFFE+PRF in scar management while highlighting the necessity for additional research into the molecular mechanisms governing adipogenesis and collagen remodeling. This study indicates that the combination of CEFFE and PRF may be an effective approach for enhancing hypertrophic scar management and facilitating skin regeneration [37].

The 2024 study examined the efficacy of cell‐free adipose tissue extracts (ATEs) as a therapeutic agent for rosacea. The study employed an in vivo rosacea‐like model in 6‐week‐old BALB/c mice and in vitro experiments with HaCaT keratinocytes to assess the effects of ATEs on inflammation, telangiectasia, and skin barrier integrity, emphasizing their regulation of Transient Receptor Potential Vanilloid 1 (TRPV1). Fat tissue obtained through liposuction was used to make ATEs. The tissue was washed, centrifuged, emulsified, filtered, and then stored at −20°C. Capsaicin (CAP) was used to stimulate HaCaT cells in vitro and ATEs to see how they affected TRPV1 expression, calcium influx, and the release of inflammatory mediators. In vivo, LL‐37 injections caused mice to show symptoms similar to rosacea, which were then treated with ATEs, CAP, capsazepine (CPZ), or saline. The research demonstrated that ATEs markedly diminished TRPV1 expression, intracellular calcium influx, and the secretion of inflammatory cytokines (KLK5, IL‐6, IL‐8, and TNF‐*α*) in CAP‐stimulated cells. ATEs also reduced erythema, TEWL, mast cell infiltration, and vascular hyperplasia in vivo, which helped with rosacea symptoms. Histological examination demonstrated that ATEs diminished epidermal thickening and telangiectasia, decreased mast cell infiltration, and downregulated TRPV1 expression in cutaneous tissue. These results indicate that ATEs may modulate inflammation and enhance skin barrier function, potentially rendering them an efficacious treatment for rosacea. The study indicated the necessity for additional research to validate the effects on telangiectasia, thoroughly clarify the mechanisms of action, and delineate the roles of specific growth factors within ATEs [38].

The 2019 study assessed the therapeutic efficacy of CEFFE obtained from human adipose tissue in enhancing skin flap viability. The study examined CEFFE′s proangiogenic effects and its capacity to improve tissue survival under ischemic conditions through in vitro experiments utilizing HUVECs and an in vivo rat skin flap model. CEFFE was extracted from human adipose tissue acquired through liposuction from six female volunteers and subsequently processed through washing, emulsification, and centrifugation to isolate the aqueous layer abundant in growth factors. In vitro, HUVECs treated with escalating concentrations of CEFFE demonstrated augmented proliferation, migration, and tube formation in a dose‐dependent fashion. ELISA demonstrated that CEFFE comprised proangiogenic factors, including VEGF, PDGF, bFGF, HGF, IGF‐1, TGF‐*β*1, BDNF, and GDNF. In the in vivo study, 28 Sprague Dawley rats were allocated into four groups according to CEFFE dosing (62.5, 125, and 250 *μ*L) and a control group administered PBS. Subcutaneous injections of CEFFE significantly enhanced the survival rates of skin flaps, particularly in the 125 *μ*L group, which exhibited the most pronounced improvement. Macroscopic and blood flow analyses showed that treated skin flaps had less necrosis and lived longer. Histological evaluations, encompassing HE and PECAM‐1 staining, demonstrated heightened capillary density and an elevated number of blood vessels in CEFFE‐treated groups relative to controls. Although the study yielded promising results, it underscored limitations such as a small sample size and donor variability. Additional investigation is required to ascertain the ideal CEFFE dosage, evaluate its antiapoptotic properties during early ischemia, and contrast its proangiogenic potential with that of platelet‐derived fractions. These results suggest that CEFFE is a potential therapeutic agent for promoting angiogenesis and improving survival in ischemic disorders, warranting further exploration for clinical applications [39].

The 2019 study examined the impact of CEFFE on dermal thickness, angiogenesis, and ECM synthesis. The research is aimed at investigating the regenerative potential of CEFFE by conducting in vivo experiments on 6‐week‐old BALB/c nude mice and in vitro studies using human dermal fibroblasts. CEFFE was extracted from medial thigh adipose tissue harvested through liposuction from three healthy female donors. To remove the aqueous layer, the preparation included washing, centrifugation, emulsification, and filtration. It was then stored at −20°C. In vitro, fibroblasts cultured with CEFFE exhibited enhanced proliferation and a hastened cell cycle, characterized by an increased number of cells in the S and G2/M phases. In vivo, mice were allocated into control, low‐dose (250 *μ*L CEFFE), and high‐dose (500 *μ*L CEFFE) cohorts, receiving CEFFE injections into the dorsal skin for 4 weeks. The results showed that CEFFE made the skin much thicker, with the high‐dose group seeing the greatest change. Histological examination demonstrated increased capillary density and enhanced cellular proliferation in CEFFE‐treated groups. Western blot analysis revealed heightened expression of COL‐1 and COL‐3 and diminished expression of matrix metalloproteinases (MMP‐1 and MMP‐3), coupled with increased levels of tissue inhibitors of metalloproteinases (TIMP‐1 and TIMP‐3). These results indicate that CEFFE promotes angiogenesis and the synthesis of ECM, thereby facilitating enhanced dermal structure and regeneration. The study emphasizes CEFFE′s potential for dermal thickening and skin rejuvenation; however, it is constrained by the utilization of a normal skin model instead of aged or sun‐damaged skin. The study also says that more research is needed on the long‐term safety of CEFFE, especially its possible risks of causing cancer or hypervascularity in clinical settings. This study identifies CEFFE as a potential agent for improving skin health and regeneration, necessitating additional research to refine its clinical application [40].

The 2020 study examined the therapeutic efficacy of a *γ*‐PGA hydrogel infused with CEFFE for the healing of diabetic wounds. Employing in vitro HUVECs and an in vivo diabetic wound model in male BKS‐Leprem2Cd479/Nju mice, the study investigated the effectiveness of this novel hydrogel system in promoting wound closure, angiogenesis, and collagen deposition. CEFFE was derived from human adipose tissue acquired through liposuction and subsequently processed through washing, centrifugation, emulsification, and freeze‐drying to yield a powdered extract. An EDC/NHS cross‐linking method was used to make the *γ*‐PGA hydrogel, which kept the wound moist and released CEFFE over 6 days. In vitro studies showed that the CEFFE‐*γ*‐PGA hydrogel kept CEFFE′s biological activity, which helped HUVEC tubes form and angiogenesis develop better. In vivo, diabetic mice administered the CEFFE‐*γ*‐PGA hydrogel exhibited markedly elevated wound closure rates compared to untreated controls or mice receiving *γ*‐PGA hydrogel alone. On Day 17, the CEFFE‐*γ*‐PGA group′s wound area had shrunk to only 2% of its original size. In the control group, it had shrunk to 45%, and in the *γ*‐PGA hydrogel group, it had shrunk to 18%. Histological analysis indicated that the wounds in the CEFFE‐*γ*‐PGA group were completely closed, the epidermis was forming continuously, and collagen was being deposited densely. Immunohistochemical staining revealed elevated capillary density (33/mm^2^) and the highest cell proliferation rate across all groups. This study emphasized the benefits of CEFFE as a substitute for recombinant growth factors, tackling issues like instability and elevated costs linked to conventional growth factors. More research is needed, though, to find out if the hydrogel can control inflammation and help nerves grow back. The findings indicate that CEFFE‐*γ*‐PGA hydrogel represents a promising therapeutic approach for diabetic wound healing, utilizing sustained growth factor delivery and a moist healing environment to promote tissue regeneration [41].

## 9. Systemic Applications—CEFFE in Sepsis

Beyond tissue‐specific regeneration, a 2022 study demonstrated that CEFFE may also exert protective effects in life‐threatening systemic inflammatory conditions. In this study, CEFFE was administered to septic mice across three models: LPS‐induced endotoxemia, *E. coli* bacteremia, and cecal ligation and puncture (CLP). CEFFE significantly improved survival and alleviated sepsis‐induced lung injury. Mechanistically, CEFFE selectively suppressed NLRP3 inflammasome activation—both canonical and noncanonical pathways—without broadly dampening upstream proinflammatory cytokines (IL‐6 and TNF‐*α*), thus avoiding the immunosuppressive risks associated with broad anti‐inflammatory strategies. Co‐immunoprecipitation assays revealed that CEFFE disrupted NLRP3–ASC complex assembly, blocking downstream pyroptotic cytokine release (IL‐1*β* and IL‐18). CEFFE also attenuated mitochondrial membrane permeability and mitochondrial DNA outflow triggered by NLRP3 activators. This study extends CEFFE′s scope beyond tissue repair into acute systemic disease, highlighting its potential as a precisely targeted anti‐inflammasome agent that avoids the immunosuppressive liability of conventional sepsis treatments. Further research is needed to identify the specific CEFFE components responsible for NLRP3 inhibition and to validate these findings clinically [42].

## 10. Composition and Mechanisms of Action of CEFFE

### 10.1. Growth Factors and Their Role in Wound Healing

CEFFE has a lot of growth factors in it, such as IGF‐1, PDGF, VEGF, BDNF, EGF, TGF‐*β*, HGF, bFGF, and neurotrophic factor (NT‐3). These factors are very important for the healing of wounds [20]:

• PDGF was one of the first growth factors found to help wounds heal. PDGF plays an important role in almost every stage of the wound healing process. PDGF aids the inflammatory phase by drawing essential repair cells, including neutrophils, macrophages, fibroblasts, and smooth muscle cells, to the wound site. It promotes fibroblast proliferation and aids in the synthesis of ECM, which is vital for blood vessel regeneration, granulation tissue formation, and the restoration of the epithelial layer at the wound site [43]. This multifunctionality has positioned PDGF as an essential element in the wound healing process. Both experimental and clinical investigations have underscored PDGF′s significance in the management of wound healing disorders [44]. Diabetic mice with diminished PDGF levels demonstrate delayed and compromised wound healing [45]. In contrast, excessive PDGF production correlates with hypertrophic scars and keloids, attributable to its significant influence on fibroblast proliferation and ECM synthesis [46].
• The VEGF family, especially VEGF‐A, is critical for wound healing because it helps new blood vessels grow [47]. Different types of cells, such as endothelial cells, fibroblasts, and macrophages, make VEGF. It starts angiogenesis by widening blood vessels, breaking down the basement membrane, and moving endothelial cells, which then multiply and form new capillary structures. VEGF also helps endothelial cells live longer by raising the levels of antiapoptotic proteins, which protect these cells from programmed cell death caused by stressors like TNF‐*α* and radiation. Lower levels of VEGF‐A are linked to slower healing, while higher levels of VEGF‐A are linked to faster healing and speed up healing in ischemic wounds [48].
• EGF is critical for wound healing because it helps keratinocytes and fibroblasts grow and move, which are both necessary for re‐epithelialization and tissue repair. The activity of EGF is closely related to the speeding up of early wound closure [49]. EGF binds to its receptor, EGFR, which starts signaling pathways that make cells grow and divide. This helps repair the damaged skin barrier. EGF not only boosts the activity of keratinocytes, but it also helps fibroblasts work better, which leads to the formation of granulation tissue [50]. EGF also works with other growth factors, like IGF‐1, to accelerate the growth of keratinocytes, showing how important it is for quick and effective wound healing [51].
• TGF‐*β* is a key factor in wound healing because it guides the process from inflammation to tissue repair. TGF‐*β*1 is the most studied of the three isoforms because it plays a big role in keratinocyte movement, the growth of granulation tissue, and the shrinking of wounds. After an injury, keratinocytes, platelets, macrophages, and fibroblasts quickly make TGF‐*β*1. It starts inflammation and makes fibroblasts make smooth muscle alpha‐actin, which turns them into myofibroblasts that help close the wound. TGF‐*β*1 also promotes angiogenesis by elevating VEGF expression, thereby facilitating sufficient blood circulation to the regenerating region. These actions make TGF‐*β*1 necessary for re‐epithelialization and effective tissue repair [52, 53].
• BDNF and NT‐3 are important neurotrophic and angiogenic factors that help new blood vessels grow, which is important for bringing blood back to damaged tissues. These factors stimulate endothelial cells to make new blood vessels, which increases the density of blood vessels in the wound area. They also speed up the healing of tissues by using the paracrine signaling system, which activates nearby cells, like mesenchymal stem cells, to release other important substances like VEGF and nitric oxide [54]. This chain of events not only promotes angiogenesis but also aids nerve regeneration, enhancing both the structural and functional recovery of the tissue. Its indirect angiogenic effects, along with its ability to help axons grow back, show that it could be useful in treating wounds and diabetic neuropathy [55].
• HGF is a flexible growth factor that has a big effect on the different stages of wound healing. In the first stage of inflammation, neutrophils and fibroblasts release HGF, which helps immune cells move to the wound site. It also makes blood vessels more permeable and helps the body move from the inflammatory phase to the proliferative phase by encouraging the growth and movement of keratinocytes and endothelial cells. This activity encourages angiogenesis, which is a crucial step in making new blood vessels that bring oxygen and nutrients to the tissue that is healing. In the last stage of remodeling, HGF controls the breakdown and deposition of the ECM by affecting matrix metalloproteinases. This makes sure that the tissue structure and function are restored correctly. HGF is a vital part of the process of healing wounds because it has many different functions [56, 57].
• IGF‐1 is a key growth factor that helps wounds heal. It plays a role in several important steps by encouraging important cellular processes like proliferation, migration, and survival. IGF‐1 accelerates the repair process by activating its Type I IGF receptor, which stimulates keratinocytes, macrophages, and fibroblasts. These cells are crucial for tissue regeneration. It also speeds up the production of COL‐1 and COL‐3, which makes the ECM stronger and helps wounds heal faster [58]. This growth factor also helps angiogenesis, which is important for getting nutrients and oxygen to the wound site [59]. IGF‐1 has also been shown to work better with other growth factors, which speeds up healing even more. IGF‐1 is a key factor in efficient wound healing because it helps with reepithelialization and tissue remodeling, which improves the vascularization and structural integrity of the regenerating skin [51].
• bFGF is essential for speeding up the healing of wounds and the growth of new skin because it encourages angiogenesis and draws important cells to the site of the injury. After tissue damage, bFGF causes chemotaxis, which brings monocytes, neutrophils, macrophages, and fibroblasts to the wound. This helps cells grow and form granulation tissue. This mitogenic activity boosts fibroblast activation and helps heal damaged skin. Furthermore, bFGF enhances vascularization by alleviating local ischemia, thereby facilitating sufficient oxygen and nutrient supply to the wound site. These cumulative effects render bFGF a crucial element in tissue repair and regeneration [60, 61].

## 11. Cytokines and Their Role in Wound Healing

M1 macrophages secrete various proinflammatory cytokines and mediators, including TNF‐*α* and IL‐1*β*, which are essential for attracting additional immune cells to the injury site to clear cellular debris via phagocytosis. Conversely, M2 macrophages are linked to anti‐inflammatory roles, releasing cytokines such as IL‐10 and demonstrating heightened expression of the mannose receptor (CD206), which facilitates tissue repair and the resolution of inflammation [13]. CEFFE has been shown to help macrophages move from the proinflammatory M1 state to the reparative M2 phenotype. This helps tissues heal while also reducing excessive inflammatory responses [43].

## 12. Synergism Versus Key Molecule Contributions—Deconstructing CEFFE′s Therapeutic Complexity

The inquiry into the regenerative effects of CEFFE remains a pivotal yet unresolved issue within the field. It is essential to determine whether these effects are predominantly the result of the synergistic interaction among its diverse bioactive constituents, functioning collectively as a biological “cocktail,” or if specific therapeutic outcomes can be attributed to distinct key molecules acting independently. The differentiation outlined herein carries significant ramifications for the processes of standardization, regulatory endorsement, clinical application, and the prospective development of next‐generation biologics inspired by CEFFE. The existing data indicates a complex, context‐sensitive framework wherein both synergistic interactions and the roles of critical molecules function concurrently, with the relative dominance of each mechanism fluctuating according to tissue type, disease context, and therapeutic objectives.

## 13. Evidence Supporting Synergism as the Primary Mechanism

The most persuasive evidence for synergism arises from direct experimental comparisons of whole CEFFE with individual trophic factors. Sun et al. [18] conducted a direct comparison of CEFFE with ciliary neurotrophic factor (CNTF), recognized as one of the most effective individual trophic factors for promoting axon regeneration, utilizing a murine optic nerve crush model. CEFFE demonstrated a marked enhancement in axon regeneration and RGC survival compared to CNTF administered alone. This finding constitutes the most compelling direct experimental evidence to date, indicating that the multicomponent composition of CEFFE offers therapeutic advantages that surpass those of any individual factor. The authors indicated that the observed outcomes were due to CEFFE comprising a variety of proteins, which include trophic factors, inflammatory factors, and chemokines. They emphasized that successful axon regeneration necessitates a synergistic interaction among multiple neurotrophic factors, a condition that cannot be met through monotherapy [18].

Additional support for synergistic effects is derived from comparative analyses of CEFFE in conjunction with PRP. Zhang et al. [29] reported that although PRP exhibited approximately 19 times greater total protein content compared to CEFFE, no statistically significant differences were observed between the two in terms of wound closure rate, re‐epithelialization, collagen deposition, Ki67‐positive cell counts, or CD31+ vessel density (*p* > 0.05; *n* = 3 per group) in both in vitro and in vivo assessments. Both CEFFE and PRP exhibited growth factor ratios that closely resemble those observed in natural physiological conditions. The authors emphasized that this natural proportionality, rather than the absolute abundance of factors, is crucial for achieving optimal synergistic therapeutic effects when compared to the application of a single growth factor in isolation [29]. This indicates that the efficacy is not dictated by the quantity of any single factor within CEFFE but is instead influenced by the preservation of native stoichiometric ratios among various factors that replicate the wound microenvironment.

Transcriptomic and proteomic analyses provide additional evidence supporting the multifaceted nature of CEFFE′s effects. In the study conducted by Nie et al. [19], a total of 1684 differentially expressed genes were identified in ADSCs subjected to treatment with CEFFE. The analysis revealed enrichment across various angiogenic pathways concurrently, indicating a response that does not align with the mechanisms of any singular growth factor [19]. In a similar vein, Yu et al. [20] identified 56 out of 1767 proteins in CEFFE that are directly related to angiogenesis. These include myeloid‐derived growth factor (MYDGF), fibronectin, and Aquaporin‐1 (AQP1), which collectively exert paracrine effects on endothelial cell proliferation, migration, and tube formation, making it challenging to ascribe these actions to any individual molecule in isolation [20]. In the realm of IVF embryo culture, Zhou et al. [28] observed that CEFFE contains a diverse array of growth factors, including FGF‐2, EGF, LIF, G‐CSF, and VEGF. This composition mirrors the natural embryotrophic environment of the uterus, indicating that it is the collective presence of these factors, rather than any individual element, that effectively mimics the in vivo embryonic niche [28].

## 14. Evidence Supporting Key Molecule Contributions

In light of the extensive evidence supporting synergistic effects, a concurrent and expanding body of research—notably from the Shanghai Ninth People′s Hospital group—has initiated a systematic deconstruction of CEFFE to pinpoint specific molecules that are accountable for particular therapeutic actions. The strategy of “component identification” signifies a significant advancement in mechanistic precision and holds promise for pharmaceutical development.

The identification of AnxA5 as a pivotal immunomodulatory molecule within CEFFE represents the most advanced example of this approach. Employing a three‐round ion exchange chromatography fractionation strategy utilizing a Q Focurose FF column, sequential NaCl gradient elutions were conducted at pH 8.0 and 5.0. Jia et al. [11] systematically separated CEFFE into distinct fractions and evaluated the capacity of each fraction to inhibit M1 macrophage polarization within an in vitro LPS/IFN‐*γ* model. Subsequent to the third purification round, the active fraction () underwent LC‐MS/MS analysis, which revealed AnxA5 as the principal immunosuppressive protein implicated in the macrophage regulatory effects of CEFFE. AnxA5 was subsequently validated as a functional key molecule, demonstrating its ability to inhibit M1 macrophage polarization through the promotion of TLR4 internalization and lysosomal degradation via calcium‐dependent endocytosis. Additionally, it was observed to reduce ROS and alleviate cartilage damage in an osteoarthritis rat model [11]. Nevertheless, it is important to note that although AnxA5 injection exhibited effects similar to whole CEFFE in the regulation of macrophages and alleviation of pain in osteoarthritis, AnxA5 alone displayed reduced efficacy relative to whole CEFFE in terms of cartilage regeneration outcomes, specifically regarding COL‐2 expression. This finding provides direct evidence that AnxA5, while a critical component for one therapeutic function (immunomodulation), is inadequate to replicate the comprehensive regenerative capabilities of CEFFE. This observation underscores the necessity of additional molecules within CEFFE that play essential complementary roles [11].

The authors explicitly acknowledged this conclusion, indicating that the identification of antiapoptotic and prochondrogenic growth factors within CEFFE could facilitate the development of a precisely engineered combination of AnxA5, antiapoptotic proteins, and prochondrogenic factors. Such a combination may yield superior outcomes in osteoarthritis compared to the use of AnxA5 in isolation, thereby establishing a vision for a “designer CEFFE” composed of rationally selected recombinant proteins. In the investigation of diabetic wound healing, Jia et al. [11] employed multiple cycles of CEFFE protein chromatography to isolate AnxA5 as the principal anti‐inflammatory protein implicated in the wound‐healing properties of CEFFE. This study highlights AnxA5 ^′^s role in facilitating the transition of macrophages from the proinflammatory M1 phenotype to the prorepair M2 phenotype. The application of recombinant human AnxA5 within GelMA hydrogels demonstrated a notable enhancement in the healing of diabetic wounds in murine models through the modulation of inflammatory responses. However, its efficacy in promoting angiogenesis and ECM regeneration was inferior to that of whole CEFFE. This observation further substantiates the notion that distinct key molecules are responsible for specific biological functions, while other components of CEFFE contribute to complementary regenerative processes [11].

## 15. A Unified Model: Context‐Dependent Synergism With Dominant Key Molecules

In light of the existing data, we present a comprehensive conceptual framework aimed at elucidating the therapeutic mechanism of CEFFE. CEFFE functions through a hierarchical synergistic system wherein [1] the collective action of over 1700 proteins generates a biomimetic paracrine environment that concurrently addresses various aspects of tissue injury, including inflammation, apoptosis, angiogenesis, matrix remodeling, and cell proliferation, in a manner that is unattainable by any singular factor, and [2] within this collective, certain functional “lead molecules” or “dominant keys” bear a disproportionate burden of responsibility for specific therapeutic effects, such as AnxA5 for immunomodulation, VEGF for angiogenesis, BDNF for neuroprotection, and MYDGF for vascular repair. The predominant key molecules function not in isolation but in collaboration with the extensive proteome, which offers a permissive signaling environment, essential cofactors, and activation of complementary pathways. The elimination of any individual key molecule diminishes, yet does not completely eliminate, the associated therapeutic effect; conversely, the removal of the entire multicomponent formulation results in a significant reduction of the therapeutic spectrum.

This model has direct and important implications for the future development of CEFFE‐based therapies: (1) In the near term, quality control strategies should include ELISA‐based monitoring of key molecules (minimum: AnxA5, VEGF, EGF, BDNF, TGF‐*β*, and PDGF‐BB) as biomarkers of CEFFE batch potency. (2) In the medium term, systematic fractionation studies—analogous to the AnxA5 discovery workflow by Jia et al. [11]—should be applied to other therapeutic endpoints (e.g., chondrogenesis, neuroregeneration, and granulosa cell proliferation) to identify additional key molecules for each tissue application. (3) In the long term, as the key molecule panel for each application is established, rational engineering of synthetic CEFFE analogs composed of defined recombinant proteins in physiologically proportionate ratios may become feasible, enabling standardized, batch‐consistent, and scalable manufacturing—a critical step toward pharmaceutical‐grade CEFFE products [11, 13, 18, 29].

## 16. Challenges and Remaining Questions

Notwithstanding the advancements outlined previously, considerable obstacles persist in the comprehensive elucidation of the synergism‐versus‐key molecule inquiry pertaining to CEFFE. The composition of CEFFE exhibits inherent variability among donors, influenced by factors such as age, BMI, sex, harvest site, and health status. This variability results in fluctuations in the relative abundance of individual key molecules, including AnxA5, across different batches, which may lead to unpredictable alterations in the synergistic balance (Jia et al., [13]; [29]). The fractionation methodology employed for the identification of AnxA5 is characterized by its labor‐intensive nature and necessitates prior knowledge of the target functional assay, such as macrophage polarization. This complexity presents challenges for universal application across various tissue contexts, particularly in the absence of well‐established in vitro screening models. Third, certain components of CEFFE may exhibit context‐dependent effects—demonstrating therapeutic benefits in specific tissues or at particular concentrations, while potentially causing adverse effects in other contexts. Jia et al. [13] observed that BDNF and GDNF present in CEFFE, although advantageous for neuroregeneration, are linked to pain sensitization in osteoarthritis when present at elevated concentrations. This finding underscores the necessity of not presuming the therapeutic safety of individual components of CEFFE based solely on their established physiological functions. Fourth, the existing proteomic profiling of CEFFE provides a snapshot of the static protein inventory; however, it fails to elucidate dynamic posttranslational modifications, protein–protein interactions, or concentration‐dependent bioactivity thresholds. These factors are essential for a comprehensive understanding of genuine synergistic interactions at the molecular level. The resolution of these challenges necessitates the incorporation of sophisticated multiomics methodologies, encompassing proteomics, transcriptomics, and interactomics, in relation to CEFFE fractions. This approach is aimed at establishing a comprehensive systems‐level understanding of the cooperative interactions among CEFFE′s components that facilitate tissue regeneration [11, 19, 20].

## 17. Proteins and Their Role in Wound Healing

Yu et al. conducted a study that produced a thorough list of 1767 proteins found in CEFFE. Out of these, 56 proteins (MYDGF, fibronectin, AQP1, etc.) were directly linked to angiogenesis, which is an important part of healing and rebuilding tissue. Finding these proteins suggests that CEFFE will be a useful tool for treating wounds and regenerating tissue in the future [20]. Details about some of these proteins and what they do are provided below:

• MYDGF: MYDGF, a paracrine protein previously demonstrated to play a key role in cardiac repair following myocardial infarction, has recently been shown to contribute to wound healing as well [32]. In a study using a full‐thickness skin injury mouse model, GelMA‐PVAMA hydrogel loaded with MYDGF significantly accelerated wound healing. This effect was ascribed to MYDGF′s capacity to stimulate angiogenesis, thereby improving blood vessel formation, oxygen transport, and nutrient provision to the injured tissue, thus expediting and enhancing tissue repair [62].
• Fibronectin: Fibronectin is a glycoprotein that is critical for wound healing because it is a key part of the ECM and helps tissues grow back when they are damaged. Fibronectin, one of the main building blocks of the ECM, interacts with different cells to make a structural framework that is necessary for healing at all stages of the repair process [63].
• AQP1: AQP1 is a protein that forms a channel for water to flow through. It is important for both cell migration and angiogenesis, which are both important for healing wounds. AQP1 helps cells change shape and make structures like lamellipodia, which are needed for migration, by making it easier for water to move across cell membranes. Research indicates that inhibiting AQP1 considerably disrupts these processes, underscoring its significance in effective wound healing and tissue regeneration [64].
• Nucleolin: Nucleolin has a special role in angiogenesis and wound healing when it is on the surface of cells in certain situations, such as inflammation or tissue regeneration [65]. Surface nucleolin serves as a receptor for multiple ligands, such as Laminin‐1 and midkine, and operates as an adhesion molecule that modulates cell–matrix interactions and internalizes stimulatory signals for endothelial cells [66]. VEGF, a key regulator of angiogenesis, moves nucleolin from the nucleus to the cell surface, which increases the movement of endothelial cells and the formation of tubules. Both of these things are important for healing wounds by forming new blood vessels. Nucleolin′s phosphorylation at its N‐terminus and the remodeling of the cytoskeleton, which is triggered by ECM attachment, are what make this happen. These mechanisms highlight nucleolin′s essential function in regulating endothelial cell activity, facilitating angiogenesis, and aiding effective tissue repair [66].

## 18. Clinical Applications of CEFFE

The limited clinical use of adipose and ADSCs is due to their rare tumorigenicity, undesirable differentiation, allograft rejection, and challenges in quality control. CEFFE is becoming a safe, effective, and cell‐free treatment that can circumvent the problems with stem cell therapy while keeping many of the advantageous things about adipose tissue, like growth factors and cytokines that help tissue heal and grow [29, 40, 67].

A clinical trial was performed to assess the effectiveness of autologous CEFFE in treating infraorbital dark circles. The study included 11 people (9 women and 2 men) between the ages of 29 and 51 (mean age 35 ± 6.6 years). They all had five treatment sessions, with 2 weeks between each one. Three months after the first treatment, the subjects were checked on again. During every treatment session, the participants received an intradermal injection of autologous CEFFE. Both objective and subjective measurements showed that the color of the infraorbital skin had significantly (*p* < 0.05) lightened and that the wrinkles around the eyes had also gotten smaller at the 3‐month follow‐up. Moreover, adverse events such as bruising, erythema, and edema were resolved spontaneously [31].

A subsequent clinical study was performed to assess the effectiveness of autologous CEFFE in the treatment of postinflammatory hyperpigmentation. The study included 15 people, 14 of whom were women and 1 of whom was a man. They were all between the ages of 18 and 33 (mean age 22.13 ± 4.29 years). They all had five treatment sessions, with 2 weeks between each one. After the first treatment, the subjects were checked again 3, 6, and 12 months later. During every treatment session, the participants received an intradermal injection of autologous CEFFE. Both objective and subjective measures showed that the color of the lesions had significantly (*p* < 0.05) lightened and that there were fewer BSs and less TEWL at the 12‐month follow‐up. Furthermore, adverse events, such as localized bruising postinjection and stinging, were temporary and well tolerated [30].

It is crucial to recognize the constraints of the aforementioned studies, including the limited number of subjects of the same ethnicity, the absence of a control group, and the relatively brief follow‐up duration. Longer follow‐up periods and more participants in studies would help us obtain more clinically useful information.

A comprehensive overview of CEFFE′s applications, mechanisms, outcomes, and challenges across various tissue systems is provided in Table 1, synthesizing evidence from preclinical and clinical studies to highlight its regenerative potential and areas for further research.

**Table 1:** Comprehensive summary of CEFFE applications in tissue regeneration: mechanisms, outcomes, and challenges.

Tissue/organ system	Key applications & mechanisms	Example studies (years & models)	Main outcomes	Challenges & future prospects
Bone, cartilage, and tendons	Repair of osteochondral defects, tendon healing, knee osteoarthritis (OA) treatment, and prevention of bone loss.	• Deng et al. (2020, rat tissue expansion) [7]	Enhanced scaffold integration; reduced cartilage degradation and matrix damage; protected osteocytes against microgravity‐induced bone loss; restored tendon structural/mechanical strength.	Donor composition variability; batch inconsistencies; tracking unexplored neurotrophic components (BDNF/GDNF) linked to pain sensitization at high doses.
		• Ding et al. (2024, mouse/HUVEC PLGA scaffolds) [10]		
		• Jia et al. (2024, rat MIA‐induced OA/AnxA5) [11]		
	Mechanisms: Growth factors (VEGF, TGF‐*β*, PDGF) promote matrix synthesis; Annexin A5 suppresses M1 macrophage polarization and ROS via TLR4/NF‐*κ*B pathway degradation; activation of ERK/MAPK and PI3K–Akt pathways drives osteogenesis; Sost suppression derepresses Wnt/*β*‐catenin.			
		• Xu et al. (2022, mouse tail suspension bone loss) [40]		
			Clinical: In a 48‐patient trial, intra‐articular injection yielded significantly superior VAS, WOMAC, and Lysholm scores over hyaluronic acid from 6 weeks to 6 months alongside bone marrow edema reduction.	
				Future: Standardization of orthopedic protocols and long‐term printing/scaffold alignment.
		• Jia et al. (2022, rat OA model) [13]		
		• Kan et al. (2023, rat Achilles tendinopathy microneedles) [14] [14]		
		• Li et al. (2025, osteoangiogenic coupling fiber coculture) [15]		
		• Nie et al. (2025, mouse ADSC osteogenesis microtissue) [16]		
		• Zhang et al. (2025, human clinical trial vs. HA) [68]		
Intervertebral disc	Management of lumbar intervertebral disc degeneration (IVDD) and associated low back pain.	• Xu et al. (2025, in vitro NPCs & rat caudal disc puncture model) [17]	Dramatically scavenged excess intracellular ROS, cleared free ferrous ion accumulation, and halted lipid peroxidation; achieved higher disc preservation scores, restored Collagen Type 2 levels, and reduced nucleus pulposus tissue loss.	Needs dedicated, optimized intradiscal slow‐release vehicles to prevent rapid clearout.
	Mechanisms: Activates the NRF2 transcription factor to upregulate GPX4 and downregulate ACSL4, actively halting iron‐dependent oxidative ferroptosis; concurrently suppresses TNF‐*α*‐induced phosphorylation of JNK, p38, and p65 (MAPK/NF‐*κ*B pathways).			
				Future: Development of injectable hydrogel delivery systems and validation in larger animal spinal models.
Nerve	Peripheral nerve injury repair, axonal outgrowth, and central neuroprotection.	• Sun et al. (2024, mouse traumatic optic nerve crush with intravitreal delivery) [18]	Promoted robust central axon regeneration and retinal ganglion cell (RGC) survival; outperformed single‐factor ciliary neurotrophic factor (CNTF) control treatments due to a broader 146‐protein neural network engagement.	Extremely scarce human clinical data for neurological applications; difficulties separating the precise neuroprotective factor combinations from the global biological extract.
	Mechanisms: Upregulation of the axonal metabolic mTOR pathway (~1.6‐fold) combined with the suppression of the inhibitory ROCK2 barrier (~0.5‐fold); modulates retinal microglial activation and drives anti‐inflammatory tissue environments via growth factor synergy.			
		• Cross‐referenced neurotrophic vascular data [20]		
Ocular applications	Repair of severe corneal epithelial injuries and neurotrophic corneal nerve degeneration.	• Xie et al. (2025, murine laser neurodegeneration & alkali‐burn models) [24]	Significantly accelerated physical corneal wound closure and triggered cell proliferation (Ki67+); preserved corneal nerve filament density and branching complexity using noninvasive delivery.	Optimizing patient compliance and structural stabilization for topical ocular administration environments.
	Mechanisms: Operates through IL‐1*β*/IL‐18‐mediated anti‐inflammatory and antiapoptotic pathways; upregulates vital endogenous neurotrophic factors on the eye surface.			
				Future: Phase‐matched trials for human neurotrophic keratopathy using stable topical drops.
Reproductive system	Premature ovarian insufficiency (POI), fertility rescue, intrauterine adhesions (IUAs), vaginal atrophy, and assisted reproductive technologies (ARTs).	• Liu et al. (2022, aged mouse and POI models) [25]	Restored AMH, E2, and FSH serum metrics; thickened atrophied vaginal walls independently of estrogen receptor dependencies; reduced endometrial fibrosis.	Discrepancies between chemically induced animal POI vs. natural human reproductive aging.
		• Kang et al. (2022, ovariectomized mouse/human vaginal atrophy cells) [26]		
	Mechanisms: Restores microvascular networks via VEGF/bFGF; drives granulosa cell proliferation via PI3K–Akt/mTOR signaling; regulates Th1/Th2 and antifibrotic stromal environments.			Future: Expanded clinical adoption of CEFFE as a standardized, certified embryotrophic culture medium supplement in IVF clinics.
		• Xu et al. (2023, mouse IUA model with hydrogels) [27]	Clinical: Supplying CEFFE directly into IVF embryo culture media for women ≥ 35 years accelerated embryo cleavage kinetics, raised blastocyst formation, minimized apoptosis, and yielded higher live birth rates.	
		• Zhou et al. (2025, prospective clinical IVF trial) [28]		
Skin and hair regeneration	Acceleration of chronic wound healing, skin rejuvenation, atopic dermatitis (AD), hypertrophic scars, rosacea, and alopecia.	• Zhang et al. (2022, mouse wounds) [29]	Reduced diabetic wound healing time (down to 14.8 days); counteracted DHT‐induced hair follicle arrest; lowered skin barrier water loss (TEWL).	Minor clinical complaints of temporary stinging or bruising during direct microinjection; structural cytotoxicity thresholds observed if concentrations exceed 500 *μ*g/mL.
		• Cai et al. (2023, clinical hyperpigmentation) [30, 33]		
	Mechanisms: EGF/PDGF drives keratinocyte/fibroblast migration; downregulates TRPV1 sensory receptors to soothe vascular hyperplasia; balances Th1/Th2 profiles; suppresses 8‐OHdG oxidative DNA damage.	• Kang et al. (2025, clinical dark circles) [31]		
			Clinical: Reduced brown spot index, mitigated wrinkles, and raised skin elasticity across under‐eye aging cohorts.	
		• Wang et al. (2020, db/db diabetic mouse wounds) [32, 69]		
		• Deng et al. (2020, skin expansion) [7]		
		• Cai et al. (2023, mouse AGA hair loss) [30, 33]		
			Flap survival: Sustained local delivery through a Ceffe@GelMA hydrogel prevented skin flap necrosis via CD31, VEGF, and *α*‐SMA activation.	
		• Fu et al. (2024, mouse atopic dermatitis) [34]		
		• Cai et al. (2025, clinical infraorbital aging) [35]		
		• Deng et al. (2019, mouse UVB photoaging) [36]		
		• Wei et al. (2024, rabbit ear hypertrophic scars) [37]		
		• Zhou et al. (2024, mouse rosacea/TRPV1) [38]		
		• Chen et al. (2025, murine skin flap hydrogel matrix) [23]		
Muscle and vascular	Treatment of acute structural muscle trauma, peripheral ischemia recovery, and therapeutic neovascularization.	• Yu et al. (2018, mouse hindlimb ischemia model) [20]	Significantly enhanced blood flow perfusion recovery via laser Doppler monitoring; checked muscle atrophy; vastly improved fat graft volume retention and survival through early microvascular stabilization.	Short half‐life and rapid structural wash‐out of liquid extracts in high‐perfusion ischemic tissues.
		• Li et al. (2024, electrospun PDA functionalized fibers) [21]		
	Mechanisms: Paracrine expression of 56 direct proangiogenic proteins (e.g., MYDGF, fibronectin, AQP1, nucleolin) that stimulate endothelial survival, migration, and structural tube formation via PI3K–Akt.			
				Future: Advanced implementation of localized biomaterial carriers (PDA fibers, customized hydrogel matrices) to prolong tissue presentation.
		• Zheng et al. (2019, mouse macrofat graft integration) [22]		
		• Nie et al. (2024, high‐quality fat graft survival) [19]		
		• Chen et al. (2025, flap thermal vascular mapping) [23]		
Systemic applications	Management of acute, life‐threatening systemic inflammatory crises and hyperinflammatory multiorgan failure.	• Wu et al. (2022, mouse endotoxemia, bacteremia, and cecal ligation/puncture models) [42]	Protected against lethal septic shock across multiple independent baseline models; alleviated acute sepsis–induced lung injury and stabilized mitochondrial membrane potential without inducing generalized immunosuppression.	Navigating the transition from localized soft tissue repair into volatile, fast‐moving systemic bloodstream delivery architectures [cite: 1].
	Mechanisms: Selectively blocks the physical assembly of the NLRP3–ASC macromolecular complex, effectively shutting down downstream pyroptotic gasdermin release (IL‐1*β*, IL‐18) without suppressing core upstream NF‐*κ*B cytokines (IL‐6, TNF‐*α*).			
				Future: Pinpointing the isolated molecule responsible for NLRP3 disruption to develop intravenous antisepsis protocols [cite: 1].

*Note:* This table provides a complete synthesis of the key information from this review manuscript on cell‐free fat extract (CEFFE), including applications across tissue systems, underlying mechanisms (e.g., growth factors and cytokines), representative studies with years and models (preclinical animal/in vitro or clinical human), main outcomes, and challenges with future prospects. This table incorporates all relevant studies mentioned in the manuscript (Refs. ([4]; [5]; [6]; [7]; [8]; [9]; [10]; [11]; [12]; [13]; [14]; [15]; [16]; [17]; [18]; [19]; [20]; [21]; [22]; [23]; [24]; [25]; [26]; [27]; [28]; [29]; [30]), ensuring no omissions of key evidence. Data are derived from over 25 studies (2017–2025)

Abbreviations: AD, atopic dermatitis; AGA, androgenetic alopecia; AMH, anti‐Müllerian hormone; AR, androgen receptor; BDNF, brain‐derived neurotrophic factor; bFGF, basic fibroblast growth factor; COL, collagen; DHT, dihydrotestosterone; ECM, extracellular matrix; EGF, epidermal growth factor; E2, estradiol; FSH, follicle‐stimulating hormone; HGF, hepatocyte growth factor; HQF, high‐quality fat; HUVECs, human umbilical vein endothelial cells; IGF‐1, Insulin‐Like Growth Factor‐1; IL, interleukin; IUA, intrauterine adhesion; MIA, monosodium iodoacetate; NF‐*κ*B, nuclear factor kappa B; NT‐3, Neurotrophin‐3; OA, osteoarthritis; PDGF, platelet‐derived growth factor; POI, premature ovarian insufficiency; RCTs, randomized controlled trials; RGC, retinal ganglion cell; ROS, reactive oxygen species; TEWL, transepidermal water loss; TGF‐*β*, transforming growth factor‐beta; TLR4, Toll‐Like Receptor 4; TNF‐*α*, tumor necrosis factor‐alpha; TRPV1, Transient Receptor Potential Vanilloid 1; VEGF, vascular endothelial growth factor.

The existing literature on CEFFE reveals a significant deficiency in the establishment of standardized dosing protocols and cohesive frameworks for efficacy evaluation across various studies. This section is aimed at enhancing scientific rigor and informing future clinical translation by synthesizing available quantitative dosage–outcome data from all included studies. It offers a comparative analysis of CEFFE in relation to benchmark treatments and proposes a framework for unified efficacy assessment.

## 19. Evaluation of Dosage–Response Relationships in Various Tissue Types

The correlation between CEFFE concentration or dosage and therapeutic outcomes has been investigated across various tissue systems, resulting in several significant findings. In the context of diabetic wound healing, the study conducted by Wang et al. [2] elucidated a significant positive dose–response relationship. Specifically, the wound closure time was observed to decrease from 22.00 ± 2.00 days in the PBS control group to 18.00 ± 1.58 days in the CEFFElow group (administered at a dosage of 2.5 mL/kg/day for 3 days via subcutaneous injection) and further to 14.80 ± 1.09 days in the CEFFEhigh group (administered at a dosage of 2.5 mL/kg/day for 6 days; *p* < 0.01; *n* = 6). Notably, the high‐dose group exhibited the most pronounced enhancements in re‐epithelialization, collagen deposition, and capillary density [69]. In a tissue expansion rat model, Deng et al. [7] demonstrated that both low‐dose (300 *μ*g protein, subcutaneous) and high‐dose (600 *μ*g protein) CEFFE resulted in a significant increase in epidermal/dermal thickness and capillary density compared to control groups. Notably, there was no statistically significant difference observed between the two dosage levels, indicating a potential therapeutic ceiling effect where a lower dosage may suffice for applications related to tissue expansion [7].

In the investigation of skin angiogenesis and dermal thickening, Xu et al. [40] conducted a study wherein CEFFE was administered subcutaneously at low (250 *μ*L) and high (500 *μ*L) doses in nude mice over a duration of 4 weeks. The results indicated that both dosage levels significantly improved dermal thickness, capillary density, and collagen synthesis, specifically noting the upregulation of COL‐1 and COL‐3. Notably, the high‐dose group exhibited a more pronounced increase in dermal thickness, suggesting a dose‐dependent relationship with respect to structural outcomes in the skin [40]. In a study conducted by Zheng et al. [22], the effects of four different doses of CEFFE (62.5, 125, and 250 *μ*L) on proangiogenic flap survival were evaluated using a rat skin flap model. The results indicated that the group receiving 125 *μ*L demonstrated the most significant enhancement in flap survival rates and capillary density. This finding implies the existence of an optimal midrange dosage, beyond which the efficacy does not increase in a proportional manner, aligning with the characteristics of inverted U‐shaped pharmacodynamics [39].

In models of osteoarthritis, Jia et al. [13] conducted in vitro assessments of CEFFE at concentrations of 100, 250, and 500 *μ*g/mL using Raw 264 cells. In a study involving macrophages stimulated with LPS/IFN‐*γ*, it was observed that CEFFE significantly reduced the expression of COX‐2 and iNOS in a concentration‐dependent manner (*p* < 0.05; *n* = 3 per group). Furthermore, in vivo intra‐articular injections administered over an 8‐week period resulted in a progressive decline in OARSI histology scores and HE fibrosis/degeneration scores with increasing CEFFE concentration, ultimately nearing normal control levels at the highest dosage evaluated [13]. In the context of AGA, the study conducted by Cai et al. [30, 33] demonstrated that the optimal therapeutic concentration in vitro was identified as 250 *μ*g/mL CEFFE for the rescue of hDPCs from DHT stress. This concentration was found to be superior to both lower (50 *μ*g/mL) and higher (500 *μ*g/mL) concentrations. This trend was corroborated by in vivo data, which indicated that the CEFFEmiddle group exhibited no statistically significant difference in hair coverage percentage compared to the CEFFEhigh group (*p* > 0.05; *n* = 12), suggesting a phenomenon of diminishing returns beyond the middle dose [33]. In a significant finding, Deng et al. [7] demonstrated that CEFFE concentrations at 500 *μ*g/mL resulted in a reduction of HaCaT cell viability following 72 h of coincubation. This observation underscores the importance of adhering to a cytotoxic upper threshold in dosing considerations [7]. In the context of reproductive applications, Liu et al. [25] conducted an investigation involving three concentrations of CEFFE (CF‐L: 0.06 *μ*g/*μ*L; CF‐M: 0.15 *μ*g/*μ*L; and CF‐H: 0.3 *μ*g/*μ*L) administered to CTX‐injured KGN granulosa cells. The results indicated a concentration‐dependent enhancement in proliferation, mitochondrial function, and antiapoptotic effects (*p* < 0.05; *n* ≥ 3 per group). Furthermore, in vivo assessments revealed that the higher dosage (3 mg/mL, 200 *μ*L every 2 days for 2 weeks via tail vein) demonstrated superior efficacy compared to the lower dosage (1.5 *μ*g/*μ*L) across most hormonal and follicle endpoints [25].

In summary, the dosage data obtained from various tissues indicate four consistent patterns: (1) a positive dose–response observed at subcytotoxic levels, particularly in contexts such as wound healing, angiogenesis, and reproductive processes; (2) the presence of a therapeutic ceiling or saturation effect at moderate dosage levels, relevant to tissue expansion and osteoarthritis; (3) an inverted U‐shaped response curve, where a midrange dose demonstrates superior efficacy compared to both lower and higher doses, as seen in skin flap procedures and AGA; and (4) the identification of a cytotoxic threshold, beyond which cell viability is compromised, specifically noted at concentrations exceeding 500 *μ*g/mL for keratinocytes. The observed patterns highlight the essential need for tissue‐ and application‐specific optimization of dosing, providing compelling evidence against the implementation of a universal dosing protocol for CEFFE.

## 20. Comparative Evaluation: CEFFE Versus Standard Treatment Protocols

In order to contextualize the efficacy of CEFFE within clinical parameters, multiple studies have conducted direct comparisons between CEFFE and established treatment modalities. Zhang et al. [29] performed a comprehensive head‐to‐head comparison of CEFFE and PRP for wound healing, employing matched in vitro and in vivo wound models. The total protein content of PRP was found to be approximately 19 times greater than that of CEFFE. However, the growth factor profiles of both were comparable, with PRP exhibiting slightly elevated levels of bFGF, HGF, TGF‐*β*, and PDGF‐BB, while CEFFE demonstrated marginally higher levels of NT‐3. In vivo assessments indicated that wound areas in both treatment groups exhibited a significantly accelerated reduction compared to control groups from Day 1, achieving near‐complete closure by Day 12. Statistical analysis revealed no significant differences between CEFFE and PRP regarding wound closure rate, re‐epithelialization, collagen deposition (as determined by Masson staining), Ki67‐positive cell counts, or CD31+ vessel density (*p* > 0.05; *n* = 3 per group). In vitro analysis indicated that PRP exhibited a marginally superior capacity for fibroblast proliferation; however, no statistically significant differences were observed in terms of migration or tube formation when compared to CEFFE (*p* > 0.05) [29]. The data suggest that CEFFE serves as an effective alternative to PRP in the context of wound healing, offering the practical benefit of a cell‐free composition that facilitates easier long‐term storage.

A significant 2025 prospective randomized controlled clinical trial conducted by Zhang et al. [68] evaluated the efficacy of intra‐articular injection of CEFFE in comparison to HA among 48 patients diagnosed with early‐to‐mid‐stage knee osteoarthritis, classified as Kellgren–Lawrence Grade II–III. Participants assigned to the CEFFE group were administered five weekly injections, each consisting of 2 mL of CEFFE. Conversely, those in the HA group received five weekly injections of 2 mL of HA, with a concentration of 1 mL/10 mg. At the 3‐week posttreatment mark, no statistically significant differences were detected in Visual Analog Scale (VAS), Western Ontario and McMaster Universities (WOMAC) Osteoarthritis Index, or Lysholm scores (*p* > 0.05). However, from the 6‐week point onward, the CEFFE group exhibited significantly lower VAS and WOMAC scores compared to the HA group (*p* < 0.05), while Lysholm scores were significantly higher in the CEFFE group (*p* < 0.05). At the 6‐month mark, the overall clinical efficacy of CEFFE demonstrated a statistically significant superiority over HA (*p* < 0.05). Additionally, the reduction in magnetic resonance imaging (MRI)–graded subchondral bone marrow edema was notably more pronounced in the CEFFE cohort (*p* < 0.05). This study constitutes the inaugural clinical trial that directly contrasts CEFFE with a standard‐of‐care intervention for osteoarthritis, revealing that CEFFE yields a superior and more sustained clinical benefit compared to HA from 6 weeks posttreatment onward [68].

## 21. Proposed Comprehensive Efficacy Assessment Framework

The lack of uniform efficacy evaluation criteria in CEFFE studies represents a considerable limitation that hinders the ability to conduct cross‐study comparisons and meta‐analytic synthesis. In light of the literature reviewed, we propose a unified minimum dataset for future CEFFE studies encompassing various tissue types.

In the context of CEFFE studies, it is imperative to report the following core parameters: (1) CEFFE protein concentration (*μ*g/mL or *μ*g/*μ*L), determined via BCA or a comparable assay, (2) the total dose administered per subject (expressed in *μ*g or mg and volume in *μ*L or mL), (3) the route and frequency of administration, (4) the duration of follow‐up, (5) quantification of key growth factors (at a minimum: VEGF, EGF, TGF‐*β*, and PDGF‐BB) utilizing ELISA to facilitate interbatch comparisons, and (6) characteristics of the donor (age, sex, BMI, and harvest site) to account for variability in outcomes.

Tissue‐specific minimum outcome metrics should encompass the following parameters: for wound healing investigations—wound closure rate (percentage per day), duration to complete closure (days), re‐epithelialization score, Ki67+ cell density, CD31+ vessel density, and collagen deposition score (Masson staining); for cartilage and osteoarthritis assessments—VAS score, WOMAC score, OARSI histology grade, and subchondral bone MRI grade; for reproductive studies—serum AMH levels (ng/mL), FSH levels (mIU/mL), E2 levels (pg/mL), follicle count, and embryo quality score; for clinical dermatological research—GAIS, LSS, and objective colorimetry (BS index, *L* *a* *b* ^∗^ values); and for all studies—adverse event reporting utilizing standardized Common Terminology Criteria for Adverse Events (CTCAE) grading.

The implementation of these standardized metrics is anticipated to significantly enhance scientific rigor, promote comparative meta‐analyses across various tissue systems, streamline regulatory submissions, and expedite the clinical translation of CEFFE. The data presented in Table 2 delineates the dosage–response relationships and comparative analyses across the principal tissue applications of CEFFE.

**Table 2:** Summary of CEFFE dosage–response relationships, comparative efficacy data, and proposed efficacy evaluation metrics across tissue applications.

Tissue/application	Study (year)	Dose/concentration tested	Key quantitative outcome	Dose–response pattern	Comparator	Comparative result
Diabetic wound healing	Wang et al. (2020) [32, 69]	CEFFElow: 2.5 mL/kg × 3 days; CEFFEhigh: 2.5 mL/kg × 6 days (subcutaneous)	Healing time: Control 22.00 ± 2.00 days → CEFFElow 18.00 ± 1.58 days → CEFFEhigh 14.80 ± 1.09 days (*p* < 0.01; *n* = 6)	Positive dose–response	PBS control	Significant improvement vs. control at both doses; CEFFEhigh superior to CEFFElow
Skin wound healing	Zhang et al. (2022) [29]	Standardized CEFFE preparation (protein concentration matched)	No significant difference in wound closure rate, Ki67, or CD31 between CEFFE and PRP groups (*p* > 0.05; *n* = 3); PRP protein ~19× higher	Comparable to PRP	PRP	Equivalent wound healing efficacy; CEFFE advantage: Cell‐free, easier storage
Tissue expansion	Deng et al. (2020) [7]	CEFFElow: 300 *μ*g protein; CEFFEhigh: 600 *μ*g protein (subcutaneous)	Both doses significantly increased epidermal/dermal thickness and CD31+ vessel density (*p* < 0.05); no significant difference between low and high	Therapeutic ceiling effect	PBS control	Both doses effective; lower dose sufficient
Skin angiogenesis/dermal thickening	Xu et al. (2020) (Xu et al. [40]	Low: 250 *μ*L; high: 500 *μ*L (subcutaneous, 4 weeks)	Both doses enhanced COL‐1, COL‐3, capillary density; high dose produced greater dermal thickening (*p* < 0.05)	Positive dose–response	Untreated	Dose‐dependent structural improvement
Skin flap survival	Zheng et al. (2019) [22]	62.5, 125, 250 *μ*L (subcutaneous)	The 125 *μ*L group showed the greatest flap survival and capillary density improvement	Inverted U‐shaped optimum	PBS control	Middose optimal; high dose did not confer added benefit
Androgenetic alopecia	Cai et al. (2023) [30, 33]	In vitro: 50, 250, 500 *μ*g/mL; in vivo: CEFFElow, CEFFEmiddle, CEFFEhigh	250 *μ*g/mL optimal in vitro; CEFFEmiddle ≈ CEFFEhigh in vivo (hair coverage; *p* > 0.05; *n* = 12)	Inverted U‐shaped; diminishing returns above the middle dose	DHT‐only model	All CEFFE doses superior to the model group (*p* < 0.05)
Osteoarthritis (in vitro/in vivo)	Jia et al. (2022) [13]	100, 250, 500 *μ*g/mL (in vitro); in vivo scores	COX‐2/iNOS reduced concentration‐dependently (*p* < 0.05; *n* = 3); OARSI/HE scores declined with increasing dose	Positive dose–response	LPS/IFN‐*γ* model	All doses superior to the untreated model
Osteoarthritis (clinical)	[2025 RCT]	CEFFE 2 mL × 5 weekly injections (intra‐articular)	VAS/WOMAC significantly lower and Lysholm score significantly higher vs. HA at 6 weeks and 3 & 6 months (*p* < 0.05); greater BME reduction	Single‐dose regimen; superior to HA from 6 weeks onward	Hyaluronic acid (HA) 2 mL × 5 weekly	CEFFE significantly superior to HA from 6 weeks; first clinical OA head‐to‐head
Reproductive (POI)	Liu et al. (2022) (Liu et al. [25]	In vitro: CF‐L 0.06, CF‐M 0.15, CF‐H 0.3 *μ*g/*μ*L; in vivo: 200 *μ*L of 1.5 or 3 *μ*g/*μ*L	Proliferation, mitochondrial function, and antiapoptosis improved concentration‐dependently in vitro; higher in vivo dose superior on hormonal endpoints (AMH, FSH, E2)	Positive dose–response	CTX/BUS‐induced POI model	Both doses superior; higher dose shows a marginal additional benefit
Cytotoxicity threshold	Deng et al. (2020) (Deng et al. [7]; Fu et al. (2024) (Fu et al. [34]	≥ 500 *μ*g/mL (in vitro, HaCaT keratinocytes)	≥ 500 *μ*g/mL inhibits cell viability after 72 h	Upper cytotoxic threshold	Normal cell viability	Safety concern at concentrations ≥ 500 *μ*g/mL in keratinocytes

Abbreviations: AGA, androgenetic alopecia; AMH, anti‐Müllerian hormone; BME, bone marrow edema; CD31, Platelet–Endothelial Cell Adhesion Molecule 1 (endothelial marker); COL, collagen; CTX, cyclophosphamide; DHT, dihydrotestosterone; E2, estradiol; FSH, follicle‐stimulating hormone; GAIS, Global Aesthetic Improvement Scale; HA, hyaluronic acid; HaCaT, human keratinocyte cell line; hDPC, human dermal papilla cell; HE, hematoxylin–eosin; Ki67, proliferation marker; LPS, lipopolysaccharide; LSS, Likert Satisfaction Scale; MRI, magnetic resonance imaging; OARSI, Osteoarthritis Research Society International; PBS, phosphate‐buffered saline; POI, premature ovarian insufficiency; PRP, platelet‐rich plasma; RCT, randomized controlled trial; VAS, Visual Analog Scale; VEGF, vascular endothelial growth factor; WOMAC, Western Ontario and McMaster Universities Osteoarthritis Index.

## 22. Integrated Signaling Pathway Analysis Across CEFFE Tissue Applications

A comprehensive evaluation of the mechanistic data from all studies included indicates that CEFFE reliably activates a conserved array of intracellular signaling pathways—MAPK/ERK, PI3K–Akt/mTOR, NF‐*κ*B, TGF‐*β*/Smad, TLR4/NLRP3, NRF2/antioxidant response, Wnt/*β*‐catenin, and mTOR–ROCK2—across various tissue types. CEFFE operates through multiple pathways, engaging these axes in a manner that is dependent on tissue context. The data reveal three primary mechanistic themes: (1) signaling that promotes antiapoptotic survival, (2) suppression of immunomodulatory responses that are specific to the context, and (3) remodeling of the ECM and angiogenesis associated with regeneration. Table 3 presents a comprehensive summary across various tissues.

**Table 3:** Integrated signaling pathway analysis of CEFFE across tissue types—verified from full‐text evidence.

Pathway	Tissues where confirmed	CEFFE effect	Key CEFFE molecules	Full‐text evidence	Mechanistic theme
MAPK/ERK, p38, JNK	Bone; OA; IVDD; nerve (retinal)	↑ prosurvival (bone, nerve); ↓ anti‐inflammatory (OA, IVDD)	IGF‐1, TGF‐*β*, VEGF, HGF	Western blot+inhibitor [12]; qRT‐PCR [13] ; WB p‐JNK/p‐p38/p‐p65 (CEFFE‐IVDD, 2025); Western blot mTOR/ROCK2 [18]	Antiapoptotic survival; immunomodulatory suppression
PI3K–Akt/mTOR	Bone (osteogenesis); reproductive (vaginal); neural (retinal/axon); vascular/angiogenesis	↑ prosurvival, proproliferative, pro‐osteogenic	VEGF, PDGF, HGF, BDNF, IGF‐1	Western blot+transcriptomics [16]; Western blot PI3K/AKT [26]; Western blot mTOR [18]; inferred from growth factor content [20]	Core shared survival and proliferation pathway
NF‐*κ*B	OA; IVDD; skin (AD, rosacea); sepsis	↓ context‐specific; not global immunosuppression	TGF‐*β*, IGF‐1, HGF, AnxA5	qRT‐PCR/flow cytometry [13]; WB p‐p65/IF (CEFFE‐IVDD, 2025); WB+RNA‐seq [11]; ELISA [19]; [34]; [38])	Immunomodulatory suppression
TGF‐*β*/Smad	Wound healing; scar; reproductive; neural; IVDD	↑ ECM synthesis, anti‐inflammatory	TGF‐*β*1 (ELISA confirmed)	ELISA TGF‐*β* [18]; Western blot COL‐1/3, TIMP ([29]; [40]); WB MMP‐3/MMP‐13 (CEFFE‐IVDD, 2025); outcomes ([37]; [27])	ECM remodeling; regeneration
TLR4/NLRP3	OA (AnxA5‐TLR4); rosacea (TRPV1); IVDD (p‐p65); sepsis (NLRP3‐ASC)	↓ tissue‐specific DAMP‐driven inflammation	AnxA5; TRPV1 modulation; direct NLRP3 disruption	RNA‐seq+LC‐MS/MS+WB P‐p65/P‐I*κ*B*α* [11]; WB/co‐IP [19]; qRT‐PCR [38]; IF p‐p65 (CEFFE‐IVDD, 2025)	Immunomodulatory suppression
NRF2/antioxidant (GPX4, SOD, catalase, GPX1)	Skin (photoaging, AD); bone; disc (antiferroptosis via GPX4); sepsis	↑ antioxidative; antiferroptotic	Growth factors upstream of NRF2; GPX1, SOD‐1/2, catalase, GPX4 downstream	Western blot enzymes ([36]; [12]); Western blot NRF2/GPX4/ACSL4+FerroOrange/Liperfluo/DCFH‐DA staining (CEFFE‐IVDD, 2025); ROS flow cytometry/IF 8‐OHdG [34]	Antiapoptotic survival; antiferroptosis
Wnt/*β*‐catenin (via Sost suppression)	Bone	↑ indirect (Sost reduction derepresses Wnt/*β*‐catenin)	Growth factors reducing Sost in osteocytes	Western blot Sost/MMP‐13 [12]	Antiapoptotic; pro‐regenerative in bone
mTOR–ROCK2 axis	Neural (optic nerve; axon regeneration)	↑ mTOR; ↓ ROCK2	HGF, BDNF	Western blot mTOR/ROCK2 [18]	Antiapoptotic; axon regeneration

Abbreviations: ACSL4, Acyl‐CoA Synthetase Long‐Chain Family Member 4; AD, atopic dermatitis; AGA, androgenetic alopecia; AnxA5, Annexin A5; BDNF, brain‐derived neurotrophic factor; bFGF, basic fibroblast growth factor; co‐IP, co‐immunoprecipitation; COL, collagen; DAMP, damage‐associated molecular pattern; DCFH‐DA, 2 ^′^,7 ^′^‐dichlorofluorescein diacetate; ECM, extracellular matrix; ERK, extracellular signal–regulated kinase; GPX, glutathione peroxidase; HGF, hepatocyte growth factor; IF, immunofluorescence; IGF‐1, Insulin‐Like Growth Factor 1; IUA, intrauterine adhesion; IVDD, intervertebral disc degeneration; JNK, c‐Jun N‐terminal kinase; LC‐MS/MS, liquid chromatography–tandem mass spectrometry; MAPK, mitogen‐activated protein kinase; MMP, matrix metalloproteinase; mTOR, mechanistic target of rapamycin; NF‐*κ*B, nuclear factor kappa‐B; NLRP3, NOD‐Like Receptor Family Pyrin Domain‐Containing 3; NRF2, Nuclear Factor Erythroid 2–Related Factor 2; OA, osteoarthritis; PDGF, platelet‐derived growth factor; PI3K, Phosphoinositide 3‐kinase; qRT‐PCR, quantitative real‐time PCR; RGC, retinal ganglion cell; ROCK2, Rho‐Associated Protein Kinase 2; ROS, reactive oxygen species; SOD, superoxide dismutase; TGF‐*β*, transforming growth factor‐beta; TIMP, tissue inhibitor of metalloproteinase; TLR4, Toll‐Like Receptor 4; TRPV1, Transient Receptor Potential Vanilloid 1; VEGF, vascular endothelial growth factor; WB, Western blot; Wnt, wingless‐related integration site.

### 22.1. MAPK/ERK and p38

The MAPK/ERK pathway exhibits the most directly validated and contextually divergent functions in CEFFE tissue applications. In bone, CEFFE induces rapid phosphorylation of both ERK and p38 in osteocytes within 1 h of treatment, as confirmed by Western blot analysis. Specific inhibition of ERK using GDC‐0994 nearly entirely negates CEFFE′s antiapoptotic protective effect, thereby establishing ERK as the principal intracellular mediator of osteocyte survival in response to ROS stress [12]. In the context of inflammation associated with osteoarthritis, CEFFE demonstrates a dose‐dependent suppression of ROS‐driven MAPK/NF‐*κ*B coactivation. This suppression leads to a reduction in the expression of iNOS, COX‐2, TNF‐*α*, and IL‐1*β* while concurrently promoting the expression of SOX‐9 and COL‐2 in macrophages and chondrocytes [13]. In the context of IVDD, findings from Western blot and immunofluorescence analyses provide direct evidence that the upregulation of p‐JNK, p‐p38, and p‐p65 induced by TNF‐*α* is significantly reduced following CEFFE treatment. This observation indicates a concurrent inhibition of the MAPK and NF‐*κ*B signaling pathways in NPCs [17]. In the context of neural regeneration, CEFFE has been observed to upregulate mTOR by approximately 1.6‐fold while concurrently reducing ROCK2 expression to about 0.5‐fold within retinal tissue. This interaction engages a MAPK/mTOR–ROCK2 axis that facilitates axonal anabolic metabolism and alleviates a significant barrier to axon regrowth [18]. The data collectively suggest that MAPK/ERK operates as a prosurvival pathway in noninflammatory CEFFE contexts, while its selective suppression occurs as a component of an anti‐inflammatory response in degenerative disease environments. This dual contextual role is influenced by the tissue microenvironment.

### 22.2. PI3K–Akt/mTOR

The PI3K–Akt/mTOR signaling pathway is widely recognized as a fundamental mechanism governing survival and proliferation in various CEFFE tissue applications. In the realm of bone tissue engineering, the activation of the PI3K–Akt pathway in ADSCs has been substantiated through transcriptomic analysis and Western blot techniques. This activation is recognized as the principal intracellular mechanism by which CEFFE promotes osteogenic differentiation and facilitates ectopic bone formation in vivo [16]. The concentration‐dependent upregulation of PI3K and AKT protein expression in reproductive tissue has been validated through Western blot analysis in vaginal epithelial VK2/E6E7 cells. This upregulation mediates the keratinocyte proliferative and mucosal thickening effects of CEFFE, occurring independently of the estrogen receptor pathway [26]. In neural tissue, the mTOR protein exhibits an upregulation of approximately 1.6‐fold in retinal tissue subsequent to intravitreal CEFFE injection, as validated by Western blot analysis, thereby reinforcing the anabolic necessities for axon regeneration and the survival of RGCs [18]. In the context of vascular biology and angiogenesis, the activation of the PI3K–Akt signaling pathway is strongly supported by the established presence of canonical growth factors known to activate this pathway, such as VEGF, HGF, PDGF, and IGF‐1. Furthermore, numerous studies have consistently reported prosurvival effects and enhanced tube formation in endothelial cells, reinforcing these findings [20, 22, 23]. Collectively, these findings establish PI3K–Akt/mTOR as the primary common molecular mechanism that underpins the cytoprotective and proproliferative properties of CEFFE in various tissues, including bone, nerve, reproductive, and vascular systems.

### 22.3. NF‐*κ*B

CEFFE demonstrates a selective and consistent suppression of NF‐*κ*B across various inflammatory disease contexts while maintaining the integrity of global immune function—representing a mechanistically precise approach to anti‐inflammatory intervention. In osteoarthritis, TGF‐*β* and IGF‐1 present in CEFFE play a role in inhibiting NF‐*κ*B activation. This inhibition occurs through the suppression of inflammatory macrophage maturation and the direct blockade of NF‐*κ*B transcriptional targets. Consequently, there is a reduction in iNOS, COX‐2, IL‐1*β*, TNF‐*α*, and ROS, alongside the promotion of M2 macrophage polarization and the expression of cartilage‐protective SOX‐9 [13]. In the context of IVDD, findings from Western blot and immunofluorescence analyses provide direct evidence that CEFFE diminishes the expression of phosphorylated p65 protein and its nuclear translocation in NPCs stimulated by TNF‐*α*. This effect is accompanied by subsequent reductions in the mRNA expression levels of COX‐2, IL‐1*β*, and iNOS [17]. In the context of skin inflammatory disorders, the suppression of proinflammatory cytokine cascades—specifically IL‐4, IL‐13, IFN‐*γ*, IL‐6, IL‐8, and TNF‐*α*—by CEFFE has been observed in models of AD and rosacea. This suppression is correlated with the restoration of Th1/Th2 balance, a reduction in TEWL, and a decrease in mast cell infiltration [34, 38]. In the context of sepsis, CEFFE has been observed to selectively inhibit the assembly of the NLRP3 inflammasome and the subsequent release of IL‐1*β* and IL‐18 while not affecting the upstream production of NF‐*κ*B‐dependent cytokines such as IL‐6 and TNF‐*α*. This mechanism effectively prevents pyroptosis and maintains the innate immune response for bacterial clearance, representing a more focused and clinically safer anti‐inflammatory strategy compared to the generalized inhibition of NF‐*κ*B [42]. The molecular entry point for NF‐*κ*B suppression exhibits tissue specificity, as evidenced by the crosstalk between the TGF‐*β*/IGF‐1 pathways in cartilage, AnxA5‐mediated TLR4 internalization and subsequent lysosomal degradation in macrophages [11], TRPV1 suppression in skin [38], and direct disruption of the NLRP3–ASC complex in systemic inflammation [42]. These findings substantiate that the anti‐inflammatory effects of CEFFE are mechanistically precise, rather than being nonspecifically immunosuppressive.

### 22.4. TGF‐*β*/Smad

TGF‐*β*, a validated component of CEFFE, has been quantified using ELISA in various studies. It signals through the canonical pathway involving Smad2/3 phosphorylation, thereby regulating ECM synthesis, cellular differentiation, and immunomodulation in diverse tissue applications. In the context of wound healing and skin regeneration, CEFFE has been observed to consistently upregulate the expression of COL‐1, COL‐3, TIMP‐1, and TIMP‐3 while concurrently downregulating MMP‐1, MMP‐3, and MMP‐9. This pattern represents the characteristic downstream proteomic signature associated with TGF‐*β*/Smad‐mediated ECM remodeling and matrix protection, as documented in recent studies [7, 29, 32, 40]. In IVDD, CEFFE demonstrates an inhibitory effect on the expression of MMP‐3 and MMP‐13 in NPCs while simultaneously promoting the restoration of ECM synthesis. This finding aligns with the TGF‐*β*/Smad‐mediated anabolic processes observed in degenerative disc tissue [17]. In the context of scar management, the integration of CEFFE with PRF demonstrates a modulation of collagen organization while concurrently diminishing the activity of pathological fibroblasts and myofibroblasts within hypertrophic scar tissue. This phenomenon is indicative of a balanced TGF‐*β*/Smad signaling pathway that promotes physiological collagen deposition as opposed to pathological deposition [37]. In the contexts of reproduction and neurology, TGF‐*β*/Smad signaling plays a significant role in the remodeling and regeneration of endometrial stroma in models of IUA [27]. Additionally, TGF‐*β*1 present in CEFFE facilitates anti‐inflammatory axon growth activity within optic nerve tissue [18]. Although the downstream ECM and cellular effects of TGF‐*β*/Smad signaling are reliably documented across various tissue applications, the direct validation of Smad2/3 phosphorylation through Western blot analysis in CEFFE‐treated cells represents a significant mechanistic gap that warrants further investigation in future research endeavors.

### 22.5. NRF2 and the Antioxidant Response Pathway

NRF2 functions as the principal transcriptional regulator of antioxidant defense, embodying a conserved cytoprotective mechanism of CEFFE that has been validated across various tissue contexts in which oxidative stress serves as a primary driver of disease. In photoaged skin, CEFFE treatment markedly increases the expression levels of GPX1, SOD‐1, SOD‐2, and catalase in UVB‐irradiated fibroblasts and murine dorsal skin. This response reflects the downstream transcriptional signature associated with NRF2 activation, with NRF2 and Sirt1/FOXO pathways recognized as the likely upstream mediators [36]. CEFFE administration results in a decrease in ROS accumulation and osteocyte apoptosis within bone, aligning with the upregulation of antioxidant genes mediated by NRF2. Additionally, there are observed reductions in Sost (sclerostin) and MMP‐13, indicating a coordinated cytoprotective effect on osteocytes [12]. In the context of IVDD, the most direct mechanistic evidence is presented: Western blot analysis indicates that CEFFE enhances the expression of NRF2 and GPX4 proteins while concurrently decreasing ACSL4 levels in TNF‐*α*‐stimulated NPCs. Additionally, reductions in ROS, free ferrous ions, and lipid peroxidation levels are substantiated through DCFH‐DA, FerroOrange, and Liperfluo staining. This evidence suggests that CEFFE mitigates ferroptosis via the NRF2 → GPX4 → antiferroptosis pathway, with optimal proliferative and cytoprotective effects observed at a concentration of 200 *μ*g/mL [17]. In AD, CEFFE demonstrates a concentration‐dependent reduction of 8‐OHdG, a biomarker indicative of oxidative DNA damage, as well as intracellular levels of ROS, as validated through immunofluorescence and flow cytometry [34]. In the context of sepsis, CEFFE has been observed to mitigate the increases in mitochondrial membrane permeability and the outflow of mitochondrial DNA induced by NLRP3 activators. These protective effects align with the NRF2‐mediated scavenging of mitochondrial ROS and the maintenance of mitochondrial structural integrity [42]. The NRF2 → GPX4 → antiferroptosis axis identified in IVDD signifies a crucial mechanistic advancement of CEFFE′s antioxidant capabilities, encompassing the prevention of iron‐dependent programmed cell death. This finding holds extensive implications for various oxidative degenerative disorders.

### 22.6. TLR4/NLRP3 Inflammasome Pathway

The TLR4–NF‐*κ*B–NLRP3 inflammasome axis is implicated in DAMP‐mediated inflammation across various disease contexts. CEFFE exerts its effects by suppressing this pathway through tissue‐specific molecular entry points, ultimately leading to a common anti‐inflammatory outcome. In osteoarthritis, the critical molecule AnxA5 has been identified through three rounds of ion exchange chromatography fractionation and subsequently validated using LC‐MS/MS. This molecule specifically inhibits the expression of gene clusters associated with the TLR signaling pathway and the activation of NF‐*κ*B by facilitating the internalization of TLR4 and its lysosomal degradation through calcium‐dependent endocytosis, achieving an internalization efficiency of 85.54*%* ± 11.6*%* at 360 min. Western blot analysis demonstrates significant reductions in phosphorylated p65 and I*κ*B*α*, while RNA sequencing combined with Gene Set Enrichment Analysis confirms the extensive transcriptomic suppression of the TLR pathway [11]. In rosacea, TRPV1, a sensory receptor that connects neurogenic and TLR4‐mediated skin inflammation, is downregulated by CEFFE, along with downstream mediators KLK5, IL‐6, IL‐8, and TNF‐*α*. This downregulation results in a reduction of erythema, vascular hyperplasia, and mast cell infiltration [38]. In the context of IVDD, direct immunofluorescence and Western blot analyses have demonstrated a reduction in p‐p65 nuclear translocation as well as downstream inflammatory gene expression in NPCs treated with CEFFE [17]. In the context of sepsis, CEFFE has been shown to disrupt the oligomerization of NLRP3 and ASC, as confirmed through co‐immunoprecipitation techniques. This compound effectively inhibits the downstream release of IL‐1*β* and IL‐18 associated with pyroptosis while not affecting the upstream production of IL‐6 and TNF‐*α* driven by NF‐*κ*B. This indicates a selective targeting of the inflammasome assembly process, rather than a generalized suppression of innate immune responses [42]. Each tissue context possesses a unique molecular entry point—AnxA5–TLR4 endocytosis, TRPV1 modulation, direct p‐p65 suppression, or NLRP3–ASC complex disruption—all culminating in the common outcome of DAMP‐driven inflammation suppression while avoiding global immunosuppression.

### 22.7. Wnt/*β*‐Catenin Pathway Modulation Through Sost Inhibition

The interaction of Wnt/*β*‐catenin with CEFFE has been substantiated in bone via an indirect yet mechanistically significant pathway. CEFFE demonstrates a significant reduction in the expression levels of Sost within osteocytes, as evidenced by Western blot analysis [12]. Given that Sost functions as the principal endogenous inhibitor of Wnt/*β*‐catenin signaling, secreted by osteocytes to attenuate osteoblastic bone formation, its reduction through CEFFE results in the derepression of the Wnt/*β*‐catenin pathway. This subsequently enhances osteoblast activity and supports the maintenance of bone mass. This effect operates in conjunction with the direct antiapoptotic mechanism mediated by ERK/MAPK within the same bone tissue environment [12]. Considering the established involvement of Wnt/*β*‐catenin signaling in hair follicle cycling and endometrial regeneration, it is imperative that future investigations assess the potential contributions of this pathway to the effects of CEFFE in applications related to AGA and IUA.

## 23. Comprehensive Overview of Mechanistic Interactions Across Tissues

Three mechanistic themes converge to unify the diverse tissue applications of CEFFE: (1) antiapoptotic survival signaling—driven by MAPK/ERK activation in bone (confirmed by inhibitor experiments [12]); PI3K–Akt/mTOR in bone, reproductive, and neural contexts (confirmed by Western blot across multiple studies [16, 18, 26]); NRF2 → GPX4 antiferroptosis in disc, skin, and bone (confirmed by protein quantification and specialized staining [17, 34, 36]); and indirect Wnt/*β*‐catenin derepression via Sost suppression in bone [12]—(2) context‐specific immunomodulatory suppression—driven by NF‐*κ*B inhibition via TGF‐*β*/IGF‐1 crosstalk in OA [13], AnxA5–TLR4 endocytosis in macrophages [11], MAPK/NF‐*κ*B cosuppression in IVDD [17], NLRP3–ASC disruption in sepsis [42], and TRPV1 modulation in rosacea [38]—and (3) regenerative ECM remodeling and angiogenesis—driven by TGF‐*β*/Smad‐mediated collagen synthesis and MMP suppression across wound healing, scar, reproductive, and disc contexts [17, 27, 29, 37] and PI3K–Akt‐mediated endothelial survival and tube formation in vascular and flap applications [20, 23]. This framework elucidates the mechanisms by which a singular complex biological preparation yields clinically significant outcomes across a range of conditions, including sepsis, disc degeneration, corneal repair, AGA, and endometrial reconstruction, by means of tissue‐specific engagement with conserved regenerative signaling networks.

## 24. Discussion

The integration of preclinical and clinical evidence in this review highlights the diverse regenerative capabilities of CEFFE across multiple tissues, establishing it as a promising cell‐free alternative to conventional adipose‐derived therapies. The effectiveness of CEFFE is attributed to its abundant growth factors (e.g., VEGF, EGF, PDGF, TGF‐*β*, and BDNF) and cytokines, which synergistically facilitate angiogenesis, cellular proliferation, ECM synthesis, and immunomodulation through M2 macrophage polarization [13, 20]. In the context of the musculoskeletal system, preclinical evidence consistently indicates that CEFFE effectively targets three specific pathological conditions: cartilage degeneration, bone loss, and tendon deterioration. In the context of cartilage regeneration, scaffolds loaded with CEFFE demonstrate superior efficacy compared to acellular scaffolds in facilitating the deposition of cartilage‐specific ECM and enhancing vascularization. This establishes CEFFE as a promising bioactive additive for the development of 3D‐printed osteochondral constructs [10]. In osteoarthritis, the therapeutic mechanism is complex: CEFFE inhibits M1 macrophage polarization and diminishes synovial inflammation via AnxA5‐mediated TLR4 internalization and NF‐*κ*B inhibition while concurrently enhancing M2‐mediated cartilage protection—an immunomodulatory profile validated in both rat models and in vitro macrophage systems [11, 13]. A 2025 randomized controlled trial conducted with 48 knee osteoarthritis patients provided a direct comparison between intra‐articular CEFFE and HA. The results indicated that CEFFE exhibited superior efficacy starting from 6 weeks, as measured by VAS, WOMAC, and Lysholm scores. This study represents the first high‐level clinical evidence supporting the musculoskeletal efficacy of CEFFE beyond its applications in dermatology [68]. CEFFE demonstrates a protective effect on osteocytes in bone, mitigating microgravity‐induced apoptosis through the activation of the MAPK/ERK pathway and the derepression of Wnt/*β*‐catenin signaling mediated by Sost, thereby restoring bone microarchitecture in tail suspension models [12]. In ADSCs, CEFFE activates the PI3K–Akt signaling pathway, thereby enhancing the formation of osteogenic microtissues in vivo. This presents a potential strategy to address the inherent limitations associated with the osteogenic potential of adipose‐derived progenitors [16]. In the context of tendinopathy, the utilization of CEFFE encapsulated within microneedles has been shown to restore the mechanical strength of the Achilles tendon and mitigate mitochondrial dysfunction more effectively than traditional direct injection methods. This underscores the critical role of optimizing delivery systems for the musculoskeletal applications of CEFFE [14]. A recently discovered application in IVDD demonstrates enhanced relevance to musculoskeletal health: CEFFE has been shown to inhibit ferroptosis in NPCs through the activation of NRF2, leading to GPX4, while concurrently suppressing the phosphorylation of JNK, p38, and p65, thereby facilitating the restoration of ECM synthesis. This was validated through both in vitro studies and rat models of disc degeneration [17].

In the context of neural and vascular applications, CEFFE exhibits the potential to effectively address injury scenarios where therapies based on individual trophic factors have repeatedly shown suboptimal performance. In the central nervous system, intravitreal CEFFE injection demonstrates a significantly enhanced capacity for axon regeneration and RGC survival post–optic nerve crush when compared to CNTF monotherapy, which serves as the standard single‐factor positive control. This effect is attributed to the synergistic activation of 146 proteins associated with axonogenesis and neurogenesis, along with mTOR upregulation and ROCK2 suppression, which together address the multifaceted barriers to axon regrowth in the CNS [18]. In corneal tissue, the application of topical CEFFE eye drops has been shown to enhance epithelial wound closure while maintaining corneal nerve density through IL‐1*β*/IL‐18‐mediated anti‐inflammatory and antiapoptotic mechanisms. This study highlights a viable noninvasive delivery method for the neuroprotective effects of CEFFE in ocular surface disorders [24]. In the context of ischemic vascular disease, CEFFE has been shown to enhance limb blood flow recovery and facilitate angiogenesis in hindlimb ischemia models. This effect is mediated through the paracrine actions of 56 identified proangiogenic proteins, which include MYDGF, fibronectin, and AQP1 [20]. The sustained delivery mechanism utilizing GelMA hydrogel markedly enhances angiogenic outcomes by preserving CEFFE bioactivity at the targeted site for a duration of 6 days, thereby addressing the rapid clearance challenges associated with direct injection methodologies [23]. In the context of fat graft applications, CEFFE enhances the survival of HQF grafts through the promotion of angiogenesis, the reduction of apoptosis, and the activation of 1684 differentially expressed genes in ADSCs. This transcriptomic evidence underscores the critical role of the multifactorial angiogenic signature of CEFFE in facilitating sustained graft integration [19].

Reproductive applications underscore CEFFE′s function in reinstating ovarian activity and preserving endometrial integrity, evidenced by enhanced follicle counts and improved pregnancy outcomes in POI and IUA models [25–27]. The most compelling evidence pertains to skin and hair regeneration, wherein CEFFE enhances wound healing in diabetic models, alleviates photoaging, and addresses conditions such as AGA, AD, rosacea, and hyperpigmentation through antioxidant, anti‐inflammatory, and re‐epithelialization mechanisms [32–34, 36, 38]. Clinical trials, though limited, validate safety and efficacy in dermatological applications, exhibiting minimal adverse effects and high patient satisfaction [30, 31, 35].

Proteomic analyses have shown that proteins such as MYDGF, fibronectin, AQP1, and nucleolin play a role in angiogenesis and wound healing [20, 62–64, 66]. CEFFE has similar growth factor profiles to PRP, but it is safer for cells [29]. Nevertheless, challenges endure, such as donor variability affected by age and health, absence of standardized preparation protocols, and the possibility of batch inconsistencies [29, 70]. Concerns regarding immunogenicity, although alleviated by the acellular composition, necessitate additional investigation [67]. The lack of extensive RCTs constrains translational confidence, especially outside dermatology [30, 31].

CEFFE′s paracrine‐driven effects are in line with bigger trends in regenerative medicine, making it a cost‐effective and easy‐to‐get treatment [3]. Subsequent investigations must emphasize multicenter trials, refined dosing strategies, and quality control indicators (e.g., ELISA for critical factors) to fully actualize its clinical potential [13, 40].

## 25. Conclusions

CEFFE represents a promising cell‐free alternative in regenerative medicine, demonstrating efficacy in enhancing tissue expansion, reducing necrosis, promoting angiogenesis, and modulating inflammation across diverse models, including burns, osteoarthritis, nerve injury, and ovarian insufficiency. Its applications span bone and cartilage repair through scaffold integration, nerve regeneration via axon growth promotion, reproductive health by restoring fertility, and skin/hair rejuvenation in wound healing and alopecia. Underlying mechanisms involve key growth factors like PDGF, TGF‐*β*, VEGF, and cytokines that drive cell proliferation, ECM synthesis, and M2 macrophage polarization. Clinical trials indicate potential in treating hyperpigmentation and dark circles with minimal side effects. However, challenges such as donor variability, lack of standardized dosing, and limited large‐scale evidence persist. Future multicenter trials, refined protocols, and quality control measures are essential to optimize CEFFE′s therapeutic consistency and broaden its clinical adoption.

## Author Contributions

All authors made substantial contributions to the conception and design of the review and participated in drafting or revising the manuscript critically for important intellectual content. Specific roles are as follows: A.K.R. and A.M. conceptualized the study and wrote the original draft; M.A. and T.F. conducted the literature search, data curation, and formal analysis; B.A. and A.B. handled methodology, visualization, and validation; S.D. and Z.S. contributed to investigation, resources, and writing—review and editing; S.M. provided supervision, project administration, and data interpretation. A.M. served as the corresponding author and oversaw the final manuscript preparation.

## Funding

No funding was received for this manuscript.

## Disclosure

The authors gave final approval for the version to be published and agreed to be accountable for all aspects of the work.

## Ethics Statement

This is a review article synthesizing existing published literature and does not involve new human or animal subjects. Therefore, no ethical approval was required. All referenced studies complied with relevant ethical guidelines, such as the Declaration of Helsinki for human research and ARRIVE guidelines for animal studies, as reported in the original publications. The authors confirm that this review adheres to ICMJE ethical standards for biomedical publications.

## Conflicts of Interest

The authors declare no conflicts of interest.

## Data Availability

Data sharing is not applicable to this article as no new data were created or analyzed in this study. All referenced data are from published sources.
